# How Does Reviewing the Evidence Change Veterinary Surgeons’ Beliefs Regarding the Treatment of Ovine Footrot? A Quantitative and Qualitative Study

**DOI:** 10.1371/journal.pone.0064175

**Published:** 2013-05-16

**Authors:** Helen M. Higgins, Laura E. Green, Martin J. Green, Jasmeet Kaler

**Affiliations:** 1 School of Veterinary Medicine and Science, University of Nottingham, Sutton Bonington, Leicestershire, United Kingdom; 2 School of Life Sciences, University of Warwick, Coventry, West Midlands, United Kingdom; Auburn University, United States of America

## Abstract

Footrot is a widespread, infectious cause of lameness in sheep, with major economic and welfare costs. The aims of this research were: (i) to quantify how veterinary surgeons’ beliefs regarding the efficacy of two treatments for footrot changed following a review of the evidence (ii) to obtain a consensus opinion following group discussions (iii) to capture complementary qualitative data to place their beliefs within a broader clinical context. Grounded in a Bayesian statistical framework, probabilistic elicitation (roulette method) was used to quantify the beliefs of eleven veterinary surgeons during two one-day workshops. There was considerable heterogeneity in veterinary surgeons’ beliefs before they listened to a review of the evidence. After hearing the evidence, seven participants quantifiably changed their beliefs. In particular, two participants who initially believed that foot trimming with topical oxytetracycline was the better treatment, changed to entirely favour systemic and topical oxytetracycline instead. The results suggest that a substantial amount of the variation in beliefs related to differences in veterinary surgeons’ knowledge of the evidence. Although considerable differences in opinion still remained after the evidence review, with several participants having non-overlapping 95% credible intervals, both groups did achieve a consensus opinion. Two key findings from the qualitative data were: (i) veterinary surgeons believed that farmers are unlikely to actively seek advice on lameness, suggesting a proactive veterinary approach is required (ii) more attention could be given to improving the way in which veterinary advice is delivered to farmers. In summary this study has: (i) demonstrated a practical method for probabilistically quantifying how veterinary surgeons’ beliefs change (ii) revealed that the evidence that currently exists is capable of changing veterinary opinion (iii) suggested that improved transfer of research knowledge into veterinary practice is needed (iv) identified some potential obstacles to the implementation of veterinary advice by farmers.

## Introduction

The UK national flock comprises 14 million breeding ewes and the mean prevalence of lameness in ewe flocks has been estimated to be 8–10% [1], [2]. Lameness costs the UK sheep industry 24 million pounds per annum [3], and is a welfare problem. Footrot is a contagious bacterial disease caused by *Dichelobacter nodosus* and it is responsible for over 80% of lameness in sheep. In the UK, farmers have traditionally treated footrot by paring the hoof horn (foot trimming) [2] and spraying the foot with a topical antibacterial. However, evidence from recent studies suggest that treatment with a parenteral long acting antibacterial cures over 90% of cases of footrot in 3 to 10 days [4]–[6]; in contrast, only 30% of sheep treated by foot trimming recovered within 10 days [4]. Prompt treatment with systemic antibacterial therapy can reduce the flock prevalence of lameness from 6%–8% to 2% [6]. Assuming the results from this study [6] are generalizable, then if this treatment were adopted by all sheep farmers the national prevalence of lameness would fall and the welfare of sheep would be improved. The Farm Animal Welfare Council published a recommendation in 2011 that ‘the prevalence of lameness in flocks farmed in Great Britain should be reduced to 5% or less within 5 years as an interim target, and to 2% or less, (which is already possible with best practice) within 10 years’ [7].

Veterinary surgeons working in private practice are ideally placed to advise and help farmers reduce lameness in sheep. A Bayesian approach was used to assess the current diversity and strength of beliefs amongst veterinary surgeons, and to quantify how presenting a review of the current evidence base influenced their opinions. In this statistical framework, probability is defined subjectively as a personal degree of belief [8]. Specifically, we used probabilistic elicitation to capture veterinary surgeons’ clinical beliefs numerically as probability distributions. An extensive literature exists on probabilistic elicitation; it is integral to Bayesian statistics and has been applied in a wide variety of fields [9], although only a few studies have used this technique in a veterinary context. Furthermore, to the authors’ knowledge, there are currently no peer-reviewed papers that have used this method to probabilistically assess how veterinary surgeons’ beliefs change in light of a review of the evidence base.

To understand why veterinary surgeons’ beliefs alter (or why they do not) requires qualitative information to augment the quantitative methodology. Qualitative information also helps to place veterinary beliefs regarding treatment efficacy in a broader clinical context and facilitates the identification of possible obstacles to the implementation of recommendations to farmers, from a veterinary perspective. Knowing these obstacles is useful so that veterinary advice can be offered to farmers in a way that they are likely to adopt; farmers will have their own beliefs regarding treatment outcomes and are faced with the practical challenges of implementing any treatment.

The aims of this research were: (i) to use probabilistic elicitation to quantify how veterinary beliefs regarding the efficacy of two treatments for footrot changed following a review of the current evidence (ii) to obtain a consensus opinion following group discussions (iii) to capture complementary qualitative data, including advice regarding treatments for footrot in general, and approaches to the delivery of advice to farmers.

## Methods

### 1. Ethics Statement

This study was approved by the Research and Ethics Committee at the School of Veterinary Medicine and Science, University of Nottingham, UK. An information sheet was provided to each participant that detailed the research objectives and requirements, and explained that the information gathered would be anonymized and published in the peer reviewed literature. It also explained that participants could stop the task at any point without giving reason; subsequently, voluntary signed consent was obtained from each participant.

### 2. Identification and Recruitment of Veterinary Surgeons

A selection of 12 veterinary surgeons was made with the following inclusion criteria: (i) at least 2 years and less than 35 years qualified, and (ii) within 4 hours driving distance of Nottingham. Of these 12 veterinary surgeons, 6 were selected using a random number generator (software program R, version 2.10.1, [10]) from the 68 who hold the Royal College of Veterinary Surgeons (RCVS) post-graduate Certificate in Sheep Health and Production (CertSHP). These are subsequently referred to as ‘certificate holders’. The remaining 6 veterinary surgeons did not hold a CertSHP, but were acknowledged within the veterinary practice in which they worked to have a demonstrable involvement with the delivery of healthcare to sheep clients, and are referred to as ‘non-certificate holders’. To identify these subjects, a random number generator was used to select veterinary practices registered as treating sheep from the RCVS database. Selected practices were contacted by telephone and the project explained; subsequently written details of the study objectives and eligibility criteria were sent by e-mail and practices were asked to confirm if an eligible veterinary surgeon was willing to attend. Potential exclusion criteria for all participants were: (i) unavailable to attend on the relevant date (ii) unwilling/unable to travel to Nottingham (iii) uncomfortable with any aspect of the task: given the nature of the exercise, full engagement and enthusiasm for the process was important for success [9].

### 3. Definitions of Treatments for Footrot

Our hypothesis was that a diverse spectrum of clinical beliefs currently exists with respect to the efficacy of two treatments for footrot in lame ewes, both of which are currently used in practice. The first treatment we considered was intra-muscular injection of long-acting oxytetracycline antibiotic (correctly dosed for the weight of animal) and topical oxytetracycline spray, with no foot paring performed. This treatment is subsequently referred to as ‘systemic and topical oxytetracycline’. The second treatment was foot paring to remove under run horn (by a proficient and experienced person) and topical oxytetracycline spray. This treatment is subsequently referred to as ‘foot trimming and topical oxytetracycline’.

### 4. Data Collection Synopsis

The non-certificate holders attended a workshop held at the University of Nottingham on the 2^nd^ July 2012, and certificate holders attended an analogous event on the 5^th^ July 2012. We ran separate workshops for certificate and non-certificate holders to avoid the possibility that some participants might be inhibited from expressing their opinions in the group discussions if they knew that other members of the group held a CertSHP, when they themselves did not. Both workshops lasted six hours and participants were provided with an inconvenience allowance of £100 per hour (pro-rata) in recognition of the time and travel required to attend the event. Participants were met on arrival and accompanied during the day by an assistant, to avoid debate until the facilitated group discussion. During the workshops, data were collected as follows.

Each veterinary surgeon was interviewed separately for one hour by either HMH or JK. The interview was recorded. The first half of the interview captured qualitative data using a standard script, concerning: (i) characteristics of the veterinary surgeons themselves, including their current clinical ovine workload and their recent appraisal of the evidence regarding footrot (ii) their current clinical approaches to treating footrot in ewes and how it compared to their perceptions of gold standard care (iii) their approaches to monitoring clinical outcomes, and how they deliver their advice to farmers. The second half of the interview captured their beliefs concerning the difference in cure rates between the two treatments for footrot (see Section 3) as probability distributions using probabilistic elicitation. This required the participant to place chips on a laminated sheet to create a histogram that quantified their current belief (see Section 5 for details).

Once all participants had completed their individual interviews, the group listened to a 30 minute power point presented using a standard script. This provided a summary of the current peer-reviewed evidence regarding the treatment of footrot and was written by LEG/JK; selection of the content included is described in Section 6. It should be noted that some of co-authors own research (JK, LEG) was included in the review of the evidence. In recognition of a potential conflict of interest, and to avoid any possibility of inhibiting participants from critically appraising and debating the evidence presented, the power point was delivered to participants by HMH, in the absence of JK and LEG, who were also not present for the remainder of the workshop.

Immediately after this, without any discussion, each participant was presented with their own laminated sheet showing the probability distribution that they had created earlier, during their interview. Participants were asked to re-consider their clinical opinions regarding the two treatments for footrot, in light of the review of the evidence, and to express their beliefs for a second time, in the same format as previously (by placing another set of chips on a new laminated sheet) to quantify their belief after hearing the review of the evidence; if their opinions had not altered, they were asked to simply replicate their original answer. A total of 45 minutes were devoted to this task, and an information sheet summarising the content of the power point presentation and copies of the four key peer-reviewed papers it described were provided to each participant, to enable participants to further appraise the information themselves. An additional sheet was also completed; this captured qualitative information relating to why participants’ beliefs had, or had not, changed. This task was completed individually, and participants were not shown the beliefs of other members of their group.

A recorded group discussion between participants followed, lasting approximately 1.5 hours. This was facilitated by HMH using a standard script, and the group were guided to discuss, in the context of the treatment of lame ewes with footrot, their views on the following: (i) the review of the evidence base (ii) foot trimming (iii) systemic antibiotics (iv) which of the two treatments had greater efficacy. Finally, the group were asked to try and achieve a consensus opinion and to express this probabilistically (in the same format as previously), such that the final probability distribution was a reflection of the knowledge, experience and beliefs of the whole group. It was recognised that to achieve a group consensus would almost inevitably involve some degree of compromise for at least some individuals, but nevertheless it was important that all participants agreed with the group distribution. This was made clear in the standard script, and in particular participants were told: ‘If necessary we can have two or more final answers which reflect real differences in opinion within the group that cannot be resolved by simply discussing and sharing current knowledge and experience.’

The method (excluding the facilitated group discussion/elicitation) was piloted on three veterinary surgeons to ensure it was tenable, and revisions made as appropriate. Data analysis is described in Section 7.

### 5. Probabilistic Elicitation

#### 5.1 Clinical context and elicited parameter

The clinical context concerned commercial flocks containing ewes lame with footrot, uncomplicated by other conditions, and affecting one foot only. The binary outcome of interest was lame (yes/no), where lame was defined as an observable limp (of any severity) and head flicking, equivalent to a locomotion score of ≥2 on the scale most commonly employed by researchers in this field [11].

The question of interest was: which treatment (as defined in Section 3) is more effective at curing footrot, in terms of the rate of recovery from lameness? There were therefore two unknown parameters: 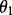
which was defined as the probability of cure in 5 days or less with systemic and topical oxytetracycline, 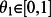, and 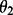 which was defined as the probability of cure in 5 days or less with foot trimming and topical oxytetracycline, 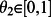. The question concerned a contrast between these two cure rates.

The time period of 5 days was chosen for two reasons. Firstly, it is likely that the recovery rate within 5 days is important with regard to limiting contagious spread and therefore has important implications for flock level control [6]. Secondly, rapid recovery is positively associated with improved ewe body condition score and lamb growth rates [12].

To quantify veterinary surgeons’ beliefs, a probability distribution was elicited for the difference in cure rates,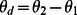,where 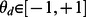, because this is a clinically intuitive scale for veterinary surgeons to use. To quantify beliefs in full regarding two unknown variables requires elicitation of the joint probability distribution however for dependent variables, as was the case here, this is a considerably more complex task [9], and was not necessary for this context.

#### 5.2 Method employed to elicit the difference in cure rates (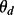)

A variety of different methods have been reported in the literature to elicit beliefs probabilistically [13]. This study employed the roulette method (also called ‘chip and bins’) because it has been shown to be feasible, valid and reliable in a clinical setting [14]. Current best practice for elicitation was followed [9], [14], which included: (i) a face-to-face interview (ii) providing examples as a training exercise (iii) use of a standardized script (see Appendix S1), (iv) a design that avoided heuristics, which are mental strategies people use to make numerical assessments in the face of uncertainty, but can introduce bias [15] (v) provision of feedback (vi) the opportunity for participants to revise their response (vii) use of simple graphical methods.

Following the general methodology of Johnson *et al* [14], participants were asked to express their belief probabilistically by indicating the weight of their belief for 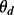 using chips each worth 0.05 probability, and placing them in discrete 5% difference intervals (the ‘bins’) across the range of 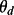. Coins, specifically 5 pence pieces, were used for the chips. Participants were given 20 chips to place, making the total probability sum to 1. Adhesive putty (Blu-Tack®, Bostik) was used to make the coins adhesive to, but easily detachable from, a laminated sheet; this is important to allow participants to revise their answers easily.

For the training exercise, 6 examples were shown to participants, each demonstrating a different belief, and the meaning of each example was explained using the standard script. The examples made abstract reference to a ‘treatment 1’ and a ‘treatment 2’ and no context was provided in order to avoid anchoring heuristics by giving a specific clinical scenario. To create familiarity with the task, the examples were created with 5 pence pieces on an almost identical laminated sheet to the one that the participants subsequently used; the only difference being that the words ‘treatment 1’ and ‘treatment 2’ were replaced by descriptions of the actual treatments.

To further minimise anchoring heuristics, the 6 examples were balanced, such that the first 2 examples illustrated beliefs that treatment 2 was definitely superior, the second 2 examples that treatment 1 was definitely superior, and the final 2 examples displayed uncertainty over which treatment was superior. Between the examples, different levels of confidence, centres of location and shapes of distribution, were illustrated. The examples are shown schematically in Appendix S2. Participants were encouraged to ask questions during this exercise. Once training was completed, the examples were placed out of sight, to avoid anchoring the participant to any of the example beliefs when considering their own answer.

The first part of the actual task involved a clarification discussion to ensure the correct clinical condition was understood by use of the term footrot. This included describing the clinical condition, and providing photographs of the clinical presentation. Clarification was also given with respect to other factors that could influence the cure rates in the first 5 days, such as the initial severity of lameness, vaccination status, and breed of ewe. Of interest was the true difference between the two treatments, i.e. any difference that is attributable to which treatment was given, once appropriate adjustments for the influence of any other factors have been made; this was made clear in the elicitation script.

Once the task was completed, the facilitator fed back to the participant the meaning of the distribution they had created in words. Participants were also encouraged to reflect upon the shape and distributions of their coins, and revise them as required. This was necessary to ensure that the distribution was a fair reflection of the participants beliefs and how much uncertainty they had in their answer.

To gather some information regarding the actual (marginal) values for 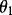
and
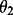
(as opposed to the difference between them), participants were also asked for an expected value and an upper and lower boundary for each parameter separately, such that they believed there was very little chance that the cure rate could fall outside of this range.

### 6. Review of the Evidence Provided during the Workshops

The details of the literature search and its results, as summarised here, were reported to the participants during the 30 minute power point presentation. In order to gather the published scientific evidence relevant to the study question, 2 databases were searched: Scopus (http://www.scopus/home.url) and MEDLINE (http://www.ncbi.nlm.nih.gov/pubmed), using a combination of one or more of the following terms: footrot, sheep, ovine, antibiotics, antibacterials, foot trimming, paring, treatment, Dichelobacter, clinical trial, randomised. This resulted in 15 primary research articles, 5 of which were discarded, either because there was no clear information regarding when sheep were monitored post treatment [16], or because they were not relevant to the treatments of interest (e.g. if the efficacy of foot trimming was only assessed when used in combination with treatments other than topical antibacterial spray) [17]–[20]. Of the remaining 10 articles, 5 were clinical trials based in Australia that assessed clinical outcome 4 to 6 weeks after treatment with systemic antibacterials [21]–[25]; these trials reported cure rates of between 80% and 99% but they did not assess foot trimming as a treatment. There were 2 UK studies that monitored the clinical outcome after 5 to 6 weeks following initial treatment with systemic antibacterials and reported cure rates above 80% but they did not assess foot trimming as a treatment [26], [27]. The remaining 3 research papers [4]–[6] were judged to be the key evidence of relevance to the question of interest, because unlike the other 7 articles, the clinical outcome was monitored at daily to weekly intervals after treatment. Furthermore, one of these papers provided information on foot trimming and topical oxytetracycline as a treatment [4]; this randomised clinical trial conducted in England, UK, involved 53 sheep in total, and reported that sheep receiving systemic antibacterials recovered faster from lameness than positive controls (odds ratio 4.92, 95% confidence interval 1.2–20.1), whereas sheep foot trimmed recovered more slowly than positive controls (odds ratio 0.05, 95% confidence interval 0.005–0.51). They also estimated the cure rate in ewes treated with long acting systemic oxytetracycline in combination with topical oxytetracycline to be 72% within 5 days, compared with a cure rate of 11% in ewes treated with foot trimming in combination with topical oxytetracycline; hence this paper supported a difference in cure rate between these two treatments in the region of 61%.

### 7. Data Analysis

#### 7.1 Quantitative data derived from probabilistic elicitation

The raw data were entered into Microsoft Excel (Version 2007, Microsoft Corp). All subsequent analysis was carried out using the software program R. It is common practice to fit parametric distributions to data originating from probabilistic elicitations, although it is widely acknowledged that this inevitably introduces some degree of imprecision, particularly as the shape inferred by the raw data may not be exactly replicated when constrained to a parametric form [15]. However as Garthwaite *et al.* [15] highlighted, ‘often a reasonable goal for elicitation is to capture the “big message” in the expert’s opinion’, and in the context of this study the precise shape of participants distributions was not a primary concern; however, the raw data overlaid with the fitted distributions are provided in Appendix S3, so the interested reader can visualise both.

Due to the scale involved, a suitable choice for the raw data was the Gaussian family, and probability density functions were fitted using numerical optimisation based on the simplex algorithm [28]_ENREF_2 to select the best fitting hyperparameters (mean and variance) by minimising the sum of the squared differences between the fitted cumulative distribution and the elicited cumulative distribution. Differences in the fitted hyperparameters before and after the review of the evidence were calculated to quantify for each participant the change in their clinical belief, whereby the mean was used as a measure of the change in central location, and the standard deviation as a change in clinical confidence. In addition, 95% Bayesian credible intervals were calculated from the fitted distributions, and used as an approximation for the interval such that participants would have assigned a 95% probability that the difference in cure rates would fall within the interval.

#### 7.2 Qualitative data from the individual interviews and facilitated group discussions

The qualitative data were transcribed and analysis involved a thematic approach [29] using NVivo qualitative data analysis software; QSR International Pty Ltd. Version 10, 2012. Transcripts were read and coded into different categories and the categories were then arranged into themes. To ensure reliability of the data the transcripts were double coded by HMH and JK. Themes were redefined where necessary after discussion to ensure there was coherence with the coded data. All the analysis of the qualitative data was inductive and was guided by the collected data.

## Results

### 1. Response Rates

For the non-certificate holders, 14 veterinary practices were contacted in total because 8 declined, giving a 43% initial response rate. Reasons given for declining were: (i) lack of enthusiasm for the task (1 practice), (ii) only a newly qualified veterinary surgeon prepared to travel to attend the workshop, but they failed the inclusion criteria with respect to years qualified (1 practice) (iii) only a newly qualified veterinary surgeon involved with delivering healthcare to sheep clients (2 practices) (iv) eligible veterinary surgeons working in the practice, but unavailable to attend on the day (four practices). For the certificate holders, 8 veterinary surgeons were contacted in total because 2 declined; in both cases, the reason given for declining was unavailable to attend on the day. For the non-certificate holders, one veterinary surgeon who confirmed their attendance, cancelled with short notice due to unpredicted clinical workload; hence this group contained only 5 veterinary surgeons, not 6.

### 2. Characteristics of Participants

Table 1 provides background information regarding the 11 participants including their current ovine workload and their appraisal of the recent evidence base with respect to footrot. All participants worked in private veterinary practice, in either England or Wales, except one veterinary surgeon who was employed by government. Numerical identifiers are subsequently used to refer to participants: non-certificate holders (1–5), certificate holders (6–11). For non-certificate holders, the median number of years qualified was 6 (range 2–20 years) compared with 22.5 years for the certificate holders (range 12–31 years). For non-certificate holders the percentage of current time spent working with sheep had a median value of 25% (range 10–25%) versus 7.5% (range 1–50%) for certificate holders. Thus, although certificate holders had more clinical experience overall, currently as a group, they reported that they were spending less of their time with sheep compared with the non-certificate holders.

**Table 1:** Characteristics of participating veterinary surgeons (n = 11).

Gender	Yearsqualified	Holder of the CertSHP?+	% of current working timespent dealingwith sheep?	Attended CPD++ events on footrot within 3 years?	Read peer-reviewedpapers on footrotwithin 3 years?	Read non peer-reviewed material on footrot within 3 years?
Male	20	No	25	No	No	No
Female	2	No	25	No	Yes	Yes
Female	5	No	10–40	Yes	Yes	No
Male	6	No	10	Yes	No	No
Male	7	No	5–30	No	No	No
Male	14	Yes	50	Yes	Yes	Yes
Male	26	Yes	10	Yes	No	Yes
Female	12	Yes	5	No	No	Yes
Male	24	Yes	<1	Yes	Yes	Yes
Female	21	Yes	5	No	Yes	Yes
Male	31	Yes	5–25	Yes	Yes	Yes

+ Certificate in Sheep Health and Production (a post-graduate qualification).

++ continuing professional development (i.e. training).

### 3. Qualitative Results from the Individual Interviews

#### 3.1 Current veterinary advice regarding the treatment of footrot in lame ewes

An open question invited participants to describe the advice they have most commonly given to commercial sheep farmers regarding treatment(s) for ewes lame with footrot, uncomplicated by other conditions of the feet. A total of 8 different pieces of advice/treatments were cited. Table 2 (column 2) gives the frequency with which each piece of advice was reported. Table 2 also contains information regarding gold standard care (see next section for details).

**Table 2:** Tally of treatments/advice recommended for footrot in lame ewes by veterinary surgeons, (n = 11).

Treatment/advice for footrot	Most commonly given advice for lame ewes in acommercial flock	Gold standard care for a single lame ewe
Proficient foot trimming	4+	8*
Topical antibacterial spray	8	10*
Systemic antibacterials	10	11
Antibacterial foot bath	0	1
Non-antibacterial foot bath	2	1
Non-steroidal anti-inflammatory pain relief	1	11
Footrot vaccination	3+	3
Remove from or improve current environment	2	8
Isolate from non-lame sheep	2	10

+ 2 vets stated they would only use vaccination as a treatment if >5% of the flock are lame.

* 2–3 vets inferred that this was case dependent.

Of the 4 participants who stated that they would usually advise foot trimming, one made reference to potential negative consequences of over-trimming and suggested that a minimal approach should be taken. Another stated that they would, if practical, advise delaying foot trimming for a few days until the infection was resolving and only trim then, if required.

An open question explored if (and how) this veterinary advice may differ, depending on the reproductive cycle. The majority of participants placed extra emphasis on minimising handling stress during very late gestation, whilst at the same time acknowledging that even heavily pregnant lame sheep must be treated promptly. One participant commented that their advice involved actively encouraging farmers to regularly monitor the flock at all times throughout the year, inferring that otherwise, in some instances, problems may only be noticed when farmers gather the flock out of necessity for key events (e.g. tupping). Another advised against footrot vaccination during the summer months to avoid the potential complication of myiasis; they also advised the use of analgesia when treating heavily pregnant lame ewes at risk of twin-lamb disease, but questioned the economic viability of analgesics at other times of the year.

Most participants stated that their advice would normally involve specifically bringing up with the farmer the question of how quickly the lame ewes need to be treated, and that they would advise treating as quickly as possible, citing limiting the spread of infection as a key reason for doing so. However some participants also suggested that treatment may be delayed in reality because of practical difficulties, and in particular identified problems associated with catching lame sheep; this issue was further explored during the group discussions (see Results Section 6.3). A minority of participants stated that they would not specifically raise the question of speed of treatment, based on an assumption that the farmer would know that treatment should be instigated immediately.

#### 3.2 Gold standard care for treating a single lame ewe

A theoretical categorical question invited participants to tick from a list of nine options the advice/treatment(s) they would consider as the initial gold standard approach to treating footrot in a *single* lame ewe, in the sense that there are no barriers to treatment such as money, or practical considerations; the frequency with which participants selected the different pieces of advice/treatments is presented in Table 2 (column 3). By comparing column 2 with column 3 (Table 2) it can be seen that the largest discrepancies between the advice usually given to commercial farmers and gold standard care for a single lame ewe were: (i) non-steroidal anti-inflammatory pain relief (ii) isolate from non-lame sheep, and (iii) removal from current environment. The most commonly cited reasons for these differences were: cost, time, labour, and/or practical considerations.

With respect to foot trimming, more participants would advise proficient trimming as part of gold standard care for a single ewe, in comparison to the advice they would usually give to commercial farmers (Table 2). A comment here was:

*“OK, with this one I think there’s a danger if you tell the farmer to trim the feet that he’ll trim it too much. So in leaving it he’s not going to do any harm. I know what I’m doing, I don’t necessarily know he knows what he’s doing, so I think it’s safer for him just to jab them and spray them, because I think that will make them better. But if I’m doing it, I know I’m not going to over trim it”.*

#### 3.3 Veterinary approaches to monitoring the clinical outcome in lame ewes

Participants were asked ‘Do you usually assume that the ewes have got better following the initial treatment, if you don’t hear anything to the contrary?’ A diversity of views was expressed. Several participants referred to trusting the farmer to report back to them if there was a poor clinical response, and this was also perceived by some to be typical practice:

*“Yes, it’s a standard vet thing, isn’t it? We just assume animals get better until you happen to see the farmer again. It’s rare that you will do a follow-up visit because the farmer won’t pay for that follow-up visit.”*

All the participants who said they would not assume a clinical recovery in the absence of any information, stressed the importance of actively establishing outcomes, and reference was also made to the need for diplomacy, as exemplified by this participant*:*

*“I think that the most important thing you can do with farmers is actually continue to probe them really, without being offensive or without accusing them of things, because you find that they don’t always do what you expect them to do. I find that’s very common, you can’t just trust them to… and this is in all walks of life, you can’t trust people to do what you tell them to do and I think if you can audit it in some way without offending them, then I think you pick up a lot of discrepancies in what you think has happened and then you can mould that into what you really want to happen”.*

One participant alluded to the fact that trusting farmers to report clinical outcomes was entirely dependent on the individual farmer:

*“There are some farmers that can be relied on to give you feedback if things aren’t going according to plan, there are some farmers that can be relied on not to. So you have to know who you’re talking to”.*

#### 3.4 Veterinary approaches to delivering advice to farmers

There were 2 open questions that explored how participants deliver their advice to farmers. All the participants agreed that they tailor the advice they give according to the action they think the farmer is likely to take in reality and the facilities and/or labour they know are available on the farm. Between them, participants provided several examples of very different situations where they would tailor their advice in the context of managing footrot (Appendix S4). Several participants acknowledged that tailoring their advice may have some negative consequences, but they also emphasised the importance of offering practical advice; some referred to perceived concerns that no action would be taken at all, if one piece of the advice they offered was considered to be impractical to the farmer, for example:

[Vet] *“So there’s no point advising stuff that you know somebody’s never going to do”.*

[Facilitator] *“And how do you make sure that you definitely know that?”.*

[Vet] *“You know the client, you get a feeling, but also you’ve potentially given that advice before and you’ve had a response….You know if you say isolate them they’re going to go pff! So you say ‘in an ideal world I would have you isolate them, I know that’s going to be difficult for you, it would be better if you could, but if you can’t…’ Which maybe gives them a get out clause…But they won’t listen to the rest of it if you add in a bit that’s so unrealistic without taking into account that you understand their system, if that makes sense”.*

### 4. The Individual and Group Elicited Probability Distributions

**Figure 1:**
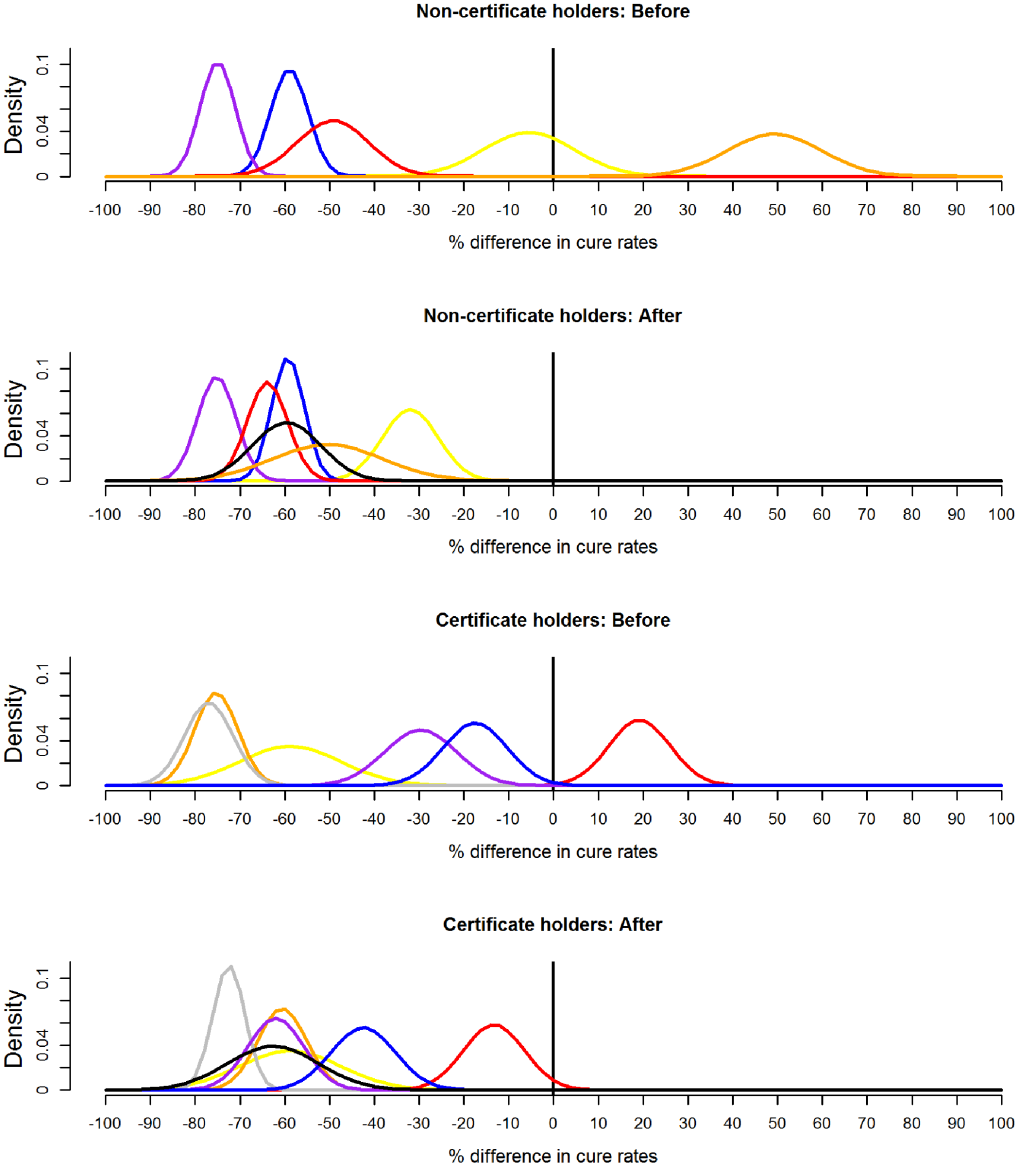
The fitted probability distributions, before and after a review of the evidence. Gaussian probability density functions fitted to the raw data for each veterinary surgeon individually, before and after a presentation of a review of the current evidence. The % difference in cure rates refers to ewes, lame with footrot, within five days of receiving either (i) systemic and topical oxytetracycline or (ii) foot trimming and topical oxytetracycline. Positive differences favour foot trimming and topical oxytetracycline, negative differences favour systemic and topical oxytetracycline. Non-certificate holders: vet 1 = yellow, vet 2 = blue, vet 3 = purple, vet 4 = red, vet 5 = orange. Certificate holders: vet 6 = yellow, vet 7 = orange, vet 8 = red, vet 9 = grey, vet 10 = purple, vet 11 = blue. The fitted probability density function to the group consensus raw data is shown in black. Values for the fitted hyperparameters (mean and variance) are listed in Table 3. The current published evidence supports a difference in cure rates of 60% in favour of systemic and topical oxytetracycline [4].

Recall that 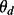
was defined as the % difference in cure rates, in ewes lame with footrot, within 5 days of receiving either (i) systemic and topical oxytetracycline or (ii) foot trimming and topical oxytetracycline. Figure 1 presents the fitted probability distributions for 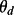, elicited individually from participants, both before and after the review of the evidence base; Table 3 details the hyperparameters of the fitted distributions and quantifies how they altered. When appraising Figure 1, it is worth recalling that the current published evidence supports a difference in cure rates in the region of 60% in favour of systemic and topical oxytetracycline [4]. Figure 1 reveals that for both groups, substantial heterogeneity existed in the beliefs of participants before the review of the evidence base, both in terms of central location and confidence. For non-certificate holders, participants’ 95% Bayesian credible intervals (together) covered a range from 83% in favour of systemic and topical oxytetracycline, to 70% in favour of foot trimming and topical oxytetracycline. For certificate holders this range was narrower, spanning 88% in favour of systemic and topical oxytetracycline to 33% favouring foot trimming and topical oxytetracycline. Furthermore, both groups contained one participant who entirely favoured foot trimming and topical oxytetracycline, in the sense that they assigned negligible probability to systemic and topical oxytetracycline offering a superior cure rate.

**Table 3:** Hyperparameters for the fitted Gaussian probability distributions.

	Fitted probability distribution, before evidence review*			Fitted probability distribution, after evidence review			Difference in fitted parameters (after-before)	
Vet ID**	Mean	Standard deviation	95% credible interval	Mean	Standard deviation	95% credible interval	Mean	Standard deviation
1	−5.5	10.2	−26,15	−32.0	6.2	−44, −20	−26.5	−4.0
2	−59.0	4.2	−67, −51	−59.3	3.6	−66, −52	−0.3	−0.6
3	−75.0	3.9	−83, −67	−75.2	4.3	−84, −67	−0.2	0.4
4	−49.3	8.0	−65, −34	−63.9	4.5	−73, −55	−14.6	−3.5
5	+49.3	10.6	29,70	−50.4	12.3	−75, −26	−99.7	1.7
6	−58.8	11.4	−81, −37	−58.8	11.4	−81, −37	0.0	0.0
7	−75.3	4.8	−85, −66	−60.6	5.5	−71, −50	14.7	0.7
8	+19.2	6.8	6,33	−13.3	6.8	−27,0	−32.5	0.0
9	−77.0	5.4	−88, −66	−72.5	3.6	−80, −66	4.5	−1.8
10	−29.7	8.0	−45, −14	−62.0	6.2	−74, −50	−32.3	−1.8
11	−17.5	7.2	−32, −4	−42.5	7.2	−57, −28	−25.0	0.0
Non-certificate holder group consensus	NA	NA	NA	−59.6	7.7	−75, −45	NA	NA
Certificate holder group consensus	NA	NA	NA	−62.6	10.2	−83, −43	NA	NA

* Positive differences favour foot trimming and topical oxytetracycline, negative differences favour systemic and topical oxytetracycline.

** Numbers 1–5 denote non-certificate holders, numbers 6–11 certificate holders.

Table 4 presents the elicited values for 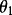
and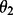, before the evidence review. It reveals that most participants expected cure rates with systemic and topical oxytetracycline to be in excess of 70%, with one notable outlier having an expectation of 20%; however there was considerably more diversity apparent over the expected cure rate with foot trimming and topical oxytetracycline. Table 4 also shows that the two participants who entirely favoured foot trimming and topical oxytetracycline initially (vet 5 and 8, Figure 1) had very different beliefs with regard to the cure rates achievable with each treatment.

**Table 4:** Elicited values for the cure rate with systemic and topical oxytetracycline (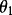) and foot trimming and topical oxytetracycline (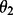).

Vet ID*	Expected value, E[ 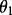 ]	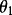 Lower to upper values	Expected value, E[ 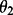 ]	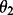 Lower to upper values	Expected difference: E[ 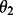 ]–E[ 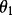 ]
1	0.75	0.55–0.85	0.65	0.50–0.75	−0.10
2	0.70	0.40–0.90	0.10	0.00–0.25	−0.60
3	0.90	0.80–0.99	0.10	0.00–0.25	−0.80
4	0.80	0.60–1.00	0.30	0.10–0.50	−0.50
5	0.20	0.00–0.50	0.50	0.30–0.60	+0.30
6	1.00	0.95–1.00	0.30	0.25–0.50	−0.70
7	0.80	0.75–0.85	0.08	0.05–0.10	+0.72
8	0.60	0.50–0.80	0.80	0.50–0.90	+0.20
9	0.80	0.60–1.00	0.05	0.00–0.10	−0.75
10	0.80	0.70–0.85	0.50	0.30–0.60	−0.30
11	0.80	0.60–1.00	0.70	0.5–0.90	−0.10

* Numbers 1–5 denote non-certificate holders, numbers 6–11 certificate holders.

Figure 1 also shows that following a review of the evidence base the heterogeneity in beliefs was demonstrably reduced, and in particular all participants now entirely favoured systemic and topical oxytetracycline. Furthermore, although the variation was greater for the non-certificate holders initially, reviewing the evidence reduced this heterogeneity relatively more in this group compared to the certificate holders. Thus for the non-certificate holders, participants’ 95% credible intervals subsequently covered a range from 20% to 84% in favour of systemic and topical oxytetracycline, whereas for certificate holders this range was wider at 0% to 81%. This was mainly due to differences in the magnitude of the change that occurred between the two participants who entirely favoured foot trimming and topical oxytetracycline at the outset; in terms of central location, vet 5 alter their belief by nearly 100% whereas vet 8 altered by 32% (Table 3).

Although three participants made only very minor adjustments to their distributions following the evidence review, only one participant (vet 6, certificate holder) did not alter their belief at all (Table 3), and interestingly the consensus for the certificate holder group was very similar to this participant’s belief.

However, even after the evidence review considerable heterogeneity remained, such that within both groups, several pairs of participants still had completely non-overlapping 95% credible intervals. Despite this, following group discussions, both groups did achieve a consensus for the difference in cure rates (Figure 1, black curves). The group consensus represents a considerable reconciliation for two participants (one in each group, vet 1 and 8), in the sense that the group 95% credible interval is non-overlapping with that of their previously expressed individual belief. The two group distributions express a very similar belief in terms of central location (means of 59.6%, versus 62.6%, Table 3), that is in keeping with the current published evidence. However the non-certificate holders expressed their consensus with slightly more confidence than the certificate holders (standard deviations of 7.7 versus 10.2, Table 3), perhaps reflecting the reduced heterogeneity amongst non-certificate holders relative to certificate holders following the evidence review.

### 5. Participants Explanations for Any Change in their Beliefs

There were 4 participants who stated that reviewing the evidence had not altered their clinical beliefs and this was reflected quantitatively (Table 3, vets 2,3,6,9). Of these, 2 confirmed that they had not changed their beliefs because they were already aware of all of the evidence provided, whilst the other 2 revealed that at least some of the information presented was new, but it concurred with their existing clinical experiences and beliefs and hence did not alter them. The remaining 7 participants stated that reviewing the evidence had altered their clinical beliefs, and again this was reflected quantitatively; of these, 2 acknowledged that they had not been previously aware of any of the evidence provided, whilst the other 5 reported to being previously aware of at least some of it. All confirmed that it was the review of the evidence base that had altered their beliefs.

### 6. Facilitated Group Discussions

Thematic analysis identified the following themes: (i) the role of foot trimming in the treatment of ewes lame with footrot (ii) veterinary involvement with lameness in sheep (iii) the practical challenges of prompt treatment (iv) elimination of footrot from some UK flocks. The debate and concepts associated with each theme are summarised below.

#### 6.1 The role of foot trimming for the treatment of lame ewes

During both group meetings, the advantages and disadvantages of foot trimming for the treatment of footrot were contested, and a diversity of views expressed; whilst some participants believed that foot trimming has no role to play in the treatment of footrot, others believed that it did, but for different reasons and to varying extents. The following extract from the certificate holders group discussion, demonstrates some of the debate:

*“I think over-paring of sheep’s feet has been proven to have disadvantages, and it does extend recovery time for lameness, but I think if trimmed carefully to expose the lesions and get air to them, it can help in recovery”.*

*“No, [be]cause that paper shows that quite clearly, even if they’re trimmed on day 6, that it makes no difference or in fact slows it down. I don’t think it has any place in the treatment of footrot”.*

*“I would disagree because when you’ve got a lot of under-running you get a situation where you have instability in the hoof and rubbing of the hoof on underlying tissue, and then I think foot trimming is very, very important, not for curing the footrot, but for stopping collateral damage if you like. I think it does matter and I think it is worth, if you can, inspecting feet sometime after they’ve been treated to see if you’ve got that problem”.*

Overall, the following arguments were put forward in favour of trimming: (i) necessitates turning the sheep over and examining the foot, and hence facilitates establishing the correct diagnosis in every individual animal (ii) it opens the lesion to the air which facilitates healing (iii) under-run or loose horn can cause mechanical damage and foot instability and needs removing (iv) if the feet are over-grown or grossly deformed they need trimming. Arguments made against recommending trimming were: (i) they may be over-trimmed, causing tissue damage which may delay recovery and/or causes additional lesions/lameness (ii) infection may be spread on equipment/hands (iii) it constitutes more, unnecessary, work for shepherds (iv) pregnant lame sheep are more likely to be treated if foot trimming is not advised because whilst some farmers may be reluctant to foot trim heavily pregnant animals for fear of inducing parturition early, they will inject them with antibiotics which is less stressful.

On both days, and throughout the individual interviews and the group discussions, the majority of participants perceived that many farmers over–trim and by doing so cause accidental damage. However the point was also raised that veterinary surgeons may not know for certain whether commercial sheep farmers trim proficiently, unless they have specifically asked the shepherd to show them how they perform this task, whilst attending the farm for some other reason:

*“You’ve clearly watched them. I have never watched. I’ve trained smallholders how to trim feet, but I’ve never watched my big [commercial] guys trimming feet.”*

One participant proposed time pressure as a reason why farmers may over-trim and also described asking farmers to demonstrate their foot trimming technique:

*“I think often farmers who are pushed for time over-trim because they take too big a bit each time, and that’s [be]cause generally I see them foot trimming… if we’re discussing lameness or we’re doing something else we grab a lame sheep, and I say, ‘Look, how would you treat this normally’ and pretty much without exception they make it bleed”.*

With respect to the concept that foot paring can facilitate the spread of infection, it was suggested that wearing gloves should be standard practice by farmers, with hands and shears disinfected between sheep, but there was general agreement that this was rarely carried out in practice:

*“It’s not rocket science, but does it happen in practice? I would say 99% of the time it doesn’t.”*

The point was also made that not all veterinary surgeons may be giving this advice, or carrying it out themselves:

*“..always have a bucket of disinfectant, disinfect your shears between every sheep, because I do not think anybody does that unless you tell them to…. Ever since I’ve started giving that advice, I’ve been doing it myself, but I’m sure I didn’t do it before…we’re probably as guilty as the farmers”.*

There was also debate over when foot trimming should be performed; some participants agreed that a few days following initial treatment with systemic antibiotics the lesions are markedly less painful and that if foot trimming is required this is the preferred time to conduct it, both for welfare reasons and because the task is easier; however it was also acknowledged that this protocol has practical implications.

An interesting comment was that veterinary support for foot trimming as a treatment for lameness in sheep may have been influenced to some degree by the fact that foot trimming plays a major role in treating lameness in dairy cattle. However it was also noted that the aetiologies of lameness in the two species are very different; a substantial proportion of lameness in cattle is related to claw horn disease whereas the majority in sheep is infectious in origin. Other issues raised during the workshops were: (i) whilst para-professional cattle foot trimmers are now recognised in the UK through a national association, with competence established by obtaining qualifications, the equivalent does not exist for sheep farmers and their para-professionals and there is scope for development in this area (ii) some farmers who have traditionally treated footrot by trimming may be difficult to convince to change this habit; settling on a compromise whereby they trim only very loose horn may be required, at least in the short term (iii) some participants commented that their advice regarding the treatment of footrot had changed in recent years towards either recommending not to foot trim, or only to trim very loose horn (iv) there was general agreement amongst certificate holders that their advice on *routine* foot trimming has changed in recent years, with a move towards not advising this practice.

#### 6.2 Veterinary involvement with lameness in sheep

During discussions, regular references were made to how much work and anxiety is created by lame sheep for farmers, as well as how widespread the problem is:

*“The problem of footrot in ewes specifically is not a small problem in clinical practice and in sheep farming, it’s an absolutely massive problem, it costs a lot of resources on a lot of sheep farms, it causes a lot of welfare problems. I think it’s a big target to aim for and that makes it doubly worth trying to improve anything to do with the treatment and control and possible eradication.”*

It was also suggested that reducing the prevalence of lameness is a key priority for many farmers. In spite of this, however, an important theme concerned how uncommon it is for veterinary surgeons to be specifically asked by farmers for lameness advice; indeed some participants reported that they usually only become involved when a member of the public has observed lame sheep and reported the farmer to an external organisation, such as the Royal Society for the Prevention of Cruelty to Animals (R.S.P.C.A). It was suggested that this is because lameness becomes tolerated by farmers, who may believe they are taking all possible action to tackle a disease that they perceive to be an inevitable constant problem. Thus farmers may only seek advice when the prevalence escalates demonstrably beyond the level they have traditionally experienced, and hence have come to accept, on their farm:

*“..but they wouldn’t necessarily bring it up, because it’s a problem that’s always there, grumbling along, and they don’t see it as a big problem unless it, I guess, balloons out of control and gets significantly worse than it was. [But that is not to say].whether or not it was a good or bad [prevalence] in the first place.”*

The point was also made that the perceived ‘acceptable’ prevalence could vary considerably between farms. Several participants emphasised the importance of taking a proactive approach, and volunteered some examples of how they themselves have done this, which included: (i) by making enquiries regarding lameness when called to attend the farm for other reasons (ii) during flock health visits (iii) through hosting farmers meetings. It was proposed that there is considerably more scope for veterinary involvement, but that without proactivity on behalf of participants, it was suggested that this was unlikely to occur:

*“I think a lot of the time you have to be proactive and when you’re on the farm or dealing with a different problem you have to be proactive and ask what’s happening, ‘How many lame sheep have you got today? Isn’t that dreadful, you’re going to have to get them all in again!’ And if you’re not proactive and if you don’t start the conversation it often doesn’t happen.”*

However, the importance of farmer levy boards (such as EBLEX in England) and the farming press, for promoting farmer awareness that lameness is a problem that should not be tolerated was highlighted and it was acknowledged that awareness is increasing. The following participant emphasised the advantages of early veterinary input:

*“I don’t think probably vets are going in until there’s a lot lame, whereas really you need to be getting in and talking to farmers preferably when there’s none lame! But you know…when there are just a few lame…the time to get in is when you can fix it, rather than trying to fix a shattered vase!”.*

In this context, several participants referred to farmers purchasing treatments for lameness from the veterinary practice without their knowledge, for example:

*“I think it would be really interesting to see how many treatments per sheep or per farm are going out to animals under our care without us knowing….people come in and buy however many bottles of Oxytet [antibiotic] and the receptionist doesn’t question it, they’ve always had however many bottles of that and they’ve had a few more this year, and we have no idea really what level of treatment is going on in terms of lameness in sheep in our practice.”*

In terms of ways to overcome this, both groups mentioned the usefulness of setting up an in-house practice monitoring system, whereby the number of treatments purchased that are likely to be deployed to treat lame sheep is regularly checked to facilitate veterinary intervention:

*“… put a note on the computer that next time they want Oxytet [antibiotic] spray they’ll need to speak to someone. We’ve done that for a few [farmers]– you try and catch them in the car park as they’re leaving with another box”.*

#### 6.3 The practical challenges of prompt treatment

During both the individual and the group discussions, several references were made to the considerable practical challenge of catching lame ewes in order to provide prompt treatment, especially for large commercial flocks with several thousand ewes grazing several hundred acres of land. In this situation, highly skilled shepherds with excellent working sheepdogs to separate individuals from the main flock along with mobile pens are a necessity, or alternatively the whole flock must be gathered. It was emphasised that gathering the entire flock can constitute a considerable amount of work, and consequently many sheep farmers only gather the flock to carry out several tasks simultaneously:

*“People rarely gather sheep to do this one thing to them; they gather sheep because they’ve got to do …gotta tail the lambs and give them their first vaccine and give them the first drench and all that lot….They try very much to group stuff like that because they haven’t got time, particularly with a big number of sheep, to go gathering them…which makes it even more difficult for treating individual lame sheep, if you say, ‘Oh that should be treated now.’ But it’s a lot of work to gather a lot of sheep to treat a couple of lame ones, and time is precious on farms, very precious.”*

Gathering the flock also carries subsequent identification difficulties, with lame sheep extremely difficult to detect; once gathered and penned, their acute stress (adrenaline) response masks their clinical signs. With the flock in close contact, the point was also made that the potential to facilitate spread of infection is increased, depending on the handling facilitates. In this context, labour was mentioned, and reported by some participants to be typically equivalent to one full-time stockperson per one thousand ewes. Thus, whilst it was acknowledged that spot treating individual lame ewes promptly is very important and is a preventive measure (by minimising spread) it was clear that for some participants the major challenge of prompt treatment for some commercial flocks made them inclined to attach extra importance to the combined use of several control measures, particularly routine vaccination, as this participant explained:

*“We’ve had issues with one large client who has 3,000 ewes and they run in open park fields of 100 or 200 acres, so unless the shepherd has a very good dog at catching things, he can’t catch individual sheep and so although the goal might be to treat them within three or four days, practically it depends on how easy it is to catch the animal, so that’s why I would advise them to vaccinate in the first place - to get the initial incidents down.”*

It was noted that this carries additional advantages in terms of the reduced use of systemic antibiotics. However it was agreed that when the flock are housed, prompt treat should not be under the influence of any practical considerations.

#### 6.4 Elimination of footrot from some individual flocks in the UK

Some attention was given during both group discussions to the issue of eliminating footrot from some individual flocks in the UK. There were some marked differences in opinion between the two groups, with non-certificate holders appearing more pessimistic; indeed some considered elimination from any flock to be extremely unlikely to be successful in the UK, primarily due to (i) the wet weather conditions (favouring environmental persistence and spread of *D. nodosus*) and (ii) the poor levels of biosecurity on UK commercial sheep farms. However certificate holders were noticeably more optimistic, indeed some participants had attempted to eliminate footrot on some of their clients’ farms, with reportedly some success; one participant reported to have eliminated footrot from approximately 40,000 sheep in total, with only a few breakdowns, usually in larger flocks (over 2,000 ewes).

There was debate over different protocols for elimination from individual flocks, including treating the entire flock once with systemic antibiotics (so called blanket use) justified on the basis that once eliminated, future use would fall to zero, versus a more conservative approach of proactively segregating and treating only lame sheep with systemic antibiotics. Irrespective of the protocol, the caveat was clearly that elimination of footrot from individual flocks should only be attempted with veterinary input and if excellent biosecurity measures are in place:

*“… But that’s because there’s no point … as number 6 says, going for the eradication if we use a lot of antibiotics and then the next week, or two weeks later, neighbour’s sheep are on the hill, poorly fenced, straying in and re-infecting.”*

Moreover, with respect to biosecurity, the possibility that wildlife may act as mechanical vectors was also mentioned. Choice of antibiotic in this context was debated, including the lack of licensed products in sheep, and it was argued that programmes that are quick and simple to implement, such as a blanket approach, are considerably more likely to be successful, especially for large flocks. The following point was also made:

*“All farmers want to eradicate lameness, but not all farmers are able to do it. I think that has to be spelled out to them”.*

## Discussion

By demonstrating considerable heterogeneity in the clinical beliefs of veterinary surgeons before a review of the evidence base, these results provide support for the hypothesis that currently a diverse spectrum of clinical beliefs exist with respect to the efficacy of systemic and topical oxytetracycline versus foot trimming and topical oxytetracycline for treating footrot. The results also showed that 7 out of the 11 participants in this study quantifiably, and in some cases markedly, altered their clinical beliefs after hearing a review of the currently published evidence. This suggests that a considerable amount of the variation in participants’ beliefs related to differences in their knowledge of the current evidence base. These findings support the notion that keeping up-to-date with the latest research findings may be difficult in veterinary practice and there are several possible reasons for this. It is recognised that the information infra-structure that underpins the translation of research findings into veterinary practice, (which includes organizations that produce systematic reviews and point-of-care decision support) is significantly underdeveloped when compared to human medicine [30]. Furthermore, the teaching of evidence-based veterinary medicine to under-graduate veterinary students has only recently gained momentum [31], and hence it is possible that some veterinary surgeons may not have fully developed the skills to search and appraise the current evidence base as efficiently as possible. Other possible obstacles include time management issues, financial constraints with respect to attending professional training events and difficulties keeping fully informed across many species.

These results have demonstrated that the current published evidence was, in this instance, of sufficient strength to sway current clinical opinion to the extent that it did convince the two participants who previously considered foot trimming and topical oxytetracycline superior, to adjust their beliefs entirely in favour of systemic and topical oxytetracycline. This is notable because it has been recognised in human medicine that an important explanation for why research may fail to alter disease management is because clinical trial results are not sufficiently strong to alter doctors’ current clinical opinions [8].

The quantitative results showed that even after a review of the evidence, considerable heterogeneity still existed amongst veterinary surgeons, both in terms of central location and confidence. The qualitative results revealed a diversity of clinical opinion concerning the role of foot trimming in the treatment of footrot, which supported the quantitative results and provided further insight. Possible reasons for the remaining heterogeneity in clinical beliefs include: (i) differences in clinical experiences per se, and how compatible the current evidence base was with a participants original beliefs (ii) differences in how the evidence base was interpreted, i.e. how ‘convincing’ it appeared (iii) differences in the perceived biological plausibility of the two treatments; given footrot is an infectious condition, systemic antibacterials as a treatment method has pharmacological credibility, whereas the biological rational for foot trimming is based on knowledge that *Dichelobacter nodosus* is an anaerobic pathogen and that paring the foot ‘lets the air in’ (iv) differences in knowledge of the non-peer reviewed literature (v) differences in personality types; in particular, some veterinary surgeons may be inherently more likely to give confident answers (narrower distributions) compared to others.

Whilst caution should be taken when attempting to make inferences to the wider veterinary community, the implications of these findings are that currently veterinary approaches to treating ovine footrot may be markedly inconsistent in practice, with potentially very different advice being given to farmers. It is proposed that more consistent advice could be achieved by improving the transfer of the latest research findings to veterinary surgeons; we suggest that far more should be done to facilitate the practice of evidence-based veterinary medicine, and research to identify the most appropriate mechanisms for rapidly disseminating species-specific research results, in an easily interpretable manner, to the relevant majority of the practising veterinary community is warranted. It should also be noted that the key research papers pertaining to the clinical question were published within the last two years, and this may explain some of the variation observed currently; eventually over time, it is likely that these findings will pervade more widely into clinical practice. However, our results also support the view that considerable heterogeneity would still be likely to remain amongst practitioners, even if knowledge transfer is improved; hence more evidence, for example in the form of a larger clinical trial, would be useful.

In terms of methodology, this study has demonstrated that using the roulette (chip and bins) elicitation approach is a practical way to quantitatively assess how veterinary surgeons’ beliefs change, in this case following a review of the current evidence, although the method could be used in any situation where formally quantifying a change in a person’s belief is required. The diversity in the elicited distributions provides support for the argument that anchoring bias was minimised; if all (or most) participants had produced very similar distributions, it would have aroused suspicion that they had been inadvertently anchored. Furthermore, the authors’ subjective perception was that veterinary surgeons found this method of elicitation straightforward, in the sense that they all appeared to quickly grasp the nature of the task and seemed comfortable with it; this was particularly important in this instance, because they had to repeat the task three times. We emphasize the usefulness of the training exercise.

In addition, the use of a combined qualitative and quantitative methodology proved fruitful to contextualize the quantitative data and identify some potential obstacles to the implementation of veterinary advice by the farming community. Perhaps most importantly, our results support the notion that despite the fact that lameness is a considerable problem, sheep farmers are unlikely to actively seek veterinary advice on this issue; hence whilst there appears to be considerably more scope for veterinary surgeons to have a positive impact, this is likely to require a proactive approach on their behalf. An important point raised by the veterinary surgeons in our sample related to the monitoring of treatments being dispensed to farmers to treat lame sheep, and the suggestion that this may be lacking in some instances. Any activity that serves to enhance veterinary involvement in lameness control would be worthwhile.

These results also support the view that more attention could usefully be given to understanding and improving veterinary approaches to *the way in which* advice is delivered to farmers. As Procter et al [32] commented, veterinary surgeons do not merely transfer research findings to farmers, rather they combine that information with their own field knowledge, in order to ‘tailor the knowledge to the circumstances of the individual farmer’ [32]. However, whilst it is clearly essential that veterinary surgeons tailor their advice to the individual farmer, nevertheless our results support the view that there may be some negative consequences of doing so, particularly if advice is tailored by veterinary surgeons’ perceived assumptions, or judgements based on failed attempts to implement control measures in the past. As Results Section 3.4 revealed, veterinary perceptions of how difficult it will be for a farmer to implement a control measure, and their concern that if they fail to acknowledge this, then no action will be taken at all, may hinder the uptake of good advice, dependent upon *how* the advice is consequently delivered. Considering alternative ways to deliver veterinary advice that do not negate the need to demonstrate an understanding of the practical challenges a farmer faces, may be useful. For example, rather than telling a farmer that it is going to be difficult for him to catch lame sheep promptly and immediately offering a (sub-optimal) alternative, broaching the issue from a positive angle at the outset could be considered. This might include beginning the conversation by highlighting the major advantages of promptly treating lame sheep and re-counting an example of another farmer who has successfully managed to do so; this could be followed by asking open questions to elicit the farmer’s thoughts on how this is will be achieved on their farm, and implicitly bringing to the discussion the supportive notion that and that *we believe* this is achievable for them. Recently, more attention has been given to the area of veterinary communication and ways to facilitate changes on farms, particularly in relation to dairy cattle [33], [34], however the same concepts apply in the context of ovine medicine.

### Conclusions

The practical importance of this study is that it has: (i) explored the current heterogeneity in veterinary beliefs regarding treatments for footrot in sheep from a sample of veterinary surgeons (ii) demonstrated a practical method for probabilistically assessing how clinical beliefs changed following a review of the evidence (iii) revealed that the current evidence that exists on the use of systemic and topical antibiotics to treat footrot in sheep is capable of changing veterinary opinion (iv) provides support for the notion that more needs to be done to improve the transfer of new evidence into clinical veterinary practice (v) identified, from a veterinary perspective, some potential obstacles to the implementation of veterinary advice by the farming community.

## Supporting Information

Appendix S1 Standard probabilistic elicitation script.
(PDF)

Appendix S2 Schematic illustration of the 6 training examples provided.
(PDF)

Appendix S3 Gaussian probability distributions fitted to the raw elicitation data.
(PDF)

Appendix S4 Examples given by veterinary surgeons of tailoring their veterinary advice.
(PDF)
