# Supplementary material for: How Does Reviewing the Evidence Change Veterinary Surgeons’ Beliefs Regarding the Treatment of Ovine Footrot? A Quantitative and Qualitative Study
Source: PLoS One. 2013 May 16;8(5):e64175. doi: 10.1371/journal.pone.0064175 (PMC3655936; doi:10.1371/journal.pone.0064175)
Supplement: Appendix S1 — Standard probabilistic elicitation script. (PDF) [file pone.0064175.s001.pdf]

Standard elicitation script: ovine footrot

For this next part, I need to show you how we would like you to give us your answer, before we begin. I will do this by talking you through six made up examples. This will take a few minutes, but please be patient even if it seems obvious we still need to go through it. If you have any questions at any point please just ask.

We are going to be interested in your opinions about the effectiveness of two different treatments, but just for now I'm not going to tell you what the treatments actually are or what they relate to, instead we are just going to call them 'treatment 1' and 'treatment 2'. You will be given a laminated sheet just like this one. As you can see there is a red line down the middle that divides the sheet into two. To the left of the red line, 'treatment 1 is better than treatment 2' and to the right of the red line, 'treatment 2 is better than treatment 1'. The sheet is divided up into intervals as marked at the bottom: 0 to 5%, 5 to 10%, etc. The intervals are the difference between the cure rates as a %. You will be given 20 chips just like these, and as you can see each 'chip' is in fact a 5 pence piece. Each chip is worth a '**5% chance**' and because you have 20 chips, in total they add up to a '100%' chance. The task involves considering each of the intervals shown, and placing chips in the intervals to indicate your **weight of belief** that the difference in the cure rates will actually fall in a given interval. The stronger you believe the actual difference will fall in a given interval the more chips you should put in that interval. If you think there is less than a 5% chance that the difference lies in a given interval don't put any chips in it.

To show you exactly how to fill the sheet in, I will go through 6 examples. If you have any questions at any point please just ask.

Example 1: Treatment 2 is **definitely a lot better** than treatment 1**.**

In this example the person believes that treatment 2 is definitely a lot better than treatment 1. They think it's most likely that with treatment 2 the cure rate will around 90% and with treatment 2 it would only be 10%. So the difference between the two cure rates would be very large: 90% - 10% = 80%. They feel strongly that this is the right answer and therefore they have put 10 chips in this interval – which is saying that they believe there is a 50% chance that the difference between the two treatments in terms of the cure rates will be about 75-80%. However, they also think it's possible that the difference could be even greater than this, and so they have put 4 chips in the 80+ interval (which is saying they think there could be a 25% chance that the difference could be greater than 80%). They also think it's possible that the difference could turn out to be a bit less than 75-80%, and so they have put 4 chips in the interval 70-75%.

Example 2: Treatment 2 is **definitely better than** treatment 1.

This second example is also showing the opinion of someone who definitely believes that Treatment 2 is better than treatment 1, but in contrast to example 1, they do not think the difference between the two treatments will be as big as 80%. Instead, they think it is most likely that the difference will turn out to be between 25-30% in favour of treatment 2 and so this is the interval in which they have put the greatest number of chips.

Please note that they might have done this because they believe most strongly that the cure rate with treatment 2 will be 70% and the cure rate with treatment 1 will be 40%, and therefore the difference between them, will be: 70% - 40%= 30%. However, it could also be because they believe most strongly that the cure rate with treatment 2 will be 90% and the cure rate with treatment 1 will be 60%, and therefore the difference between them, will be:
90% - 60% = 30%. But it doesn't matter, because in either case, they think the **difference** will turn out to be around 30% so in either case, they would put the most chips in the 25-30% interval. The key point is that this chart is only asking for your opinion about **the difference** between the two treatments, it is not saying anything directly about what you what you think the cure rate would be with each treatment separately.

Also, note that in contrast to example 1, this person has much **more uncertainty** about what the answer is, and we can see this because they have spread their chips out more horizontally. So although their 'best guess' is that there will be a 25-30% difference, they are also giving a 5% chance that there could be a much bigger difference between the two treatments, as great as 60-65%, and they are also giving a 5% chance that the difference between the two treatments might be as small as only 15-20% in favour of treatment 2.

As you can start to see now, from looking at these 2 examples, by placing the chips on the sheet, it creates a shape that represents how **plausible** you think it is that the actual difference in the cure rates (due to which treatment was given) will turn out to fall in the given intervals.

The narrower and taller the shape, the more confident you are; and the wider and lower the peak, the less certain is your opinion.

Example 3: Treatment 1 is **definitely a lot better** than treatment 2.

Here the person believes treatment 1 is definitely better than treatment 2. They are giving a 40% chance (8 chips) to the difference in the cure rates being 50-55% in favour of treatment 1. We can see that they are much more confident in their opinion compared to the person in Example 2, because their chips are not spread out over such a wide range.

Example 4: Treatment 1 is **definitely better** than treatment 2

Here the person believes treatment 1 is better than treatment 2, but whilst their 'best guess' is that the difference will be about 50-55% in favour of treatment 1, they have considerable uncertainty, because they have spread their chips out very widely indeed; thus on the one hand they are giving a 5% chance that there could be a very big difference between the cure rates (75-80%), but they are also giving a giving a 5% that the difference in the cure rates will turn out to be no more than 0-5% in favour of treatment 1.

Example 5: Not sure which treatment is better, but **favouring** treatment 1 over treatment 2.

Here the person has put some chips on either side of the 'red line'. They have put 6 chips (which is worth a '30% chance) to the right of the red line, and the remaining 14 chips (worth a total of a '70% chance) to the left of the red line. This means they are not sure if one treatment is definitely better than the other, but they are giving a 70% chance to 'treatment 2 being better than treatment 1', and a 30% chance that 'treatment 1 will be better than treatment 2'. So they are favouring treatment 2 over treatment 1. They have put the greatest number of chips in the 5-10% interval on the side that favours treatment 1 – so their 'best guess' for the answer, is that treatment 1 will provide a 5-10% improvement in cure rates over treatment 2.

Example 6: Really not sure which treatment is better! 'Sitting on the fence'!

Here the person has put half their chips to the right of the red line and the other half to the left. They believe there is a 50% chance that treatment 1 is better than treatment 2, and a 50% chance that treatment 2 is better than treatment 1! In addition, because they have not spread their chips out very much, they are also saying that, whichever treatment turns out to be better, the difference between them will not be greater than 5-10% either way.

Do you have any questions about how to fill the sheet in, or what it means? If 'no' proceed, if 'yes' further explain.

Now we will begin with the actual question.

For this, we need to check first of all that we are both talking about the same clinical condition. Please be patient, because again we have to go through this with you in full, even if it may seem obvious!

By 'footrot' we mean that there is separation of the hoof horn, that has started at the inter-digital space, and once established the sole horn and outer wall may be under-run; there is also grey oozing pus with a distinctive foul smell. The bacterium of primary importance in the pathogenesis of footrot is *Dichelobacter nodosus*.

Here are two photographs of footrot.
[*Show the vet photographs of footrot*].

For this question we are only considering **ewes** that are lame due to footrot. You can assume that a correct diagnosis has been made, that we know for certain that *Dichelobacter nodosus* is involved pathogenically, and the clinical presentation is **not** complicated by other conditions of the feet.

Do you understand the condition we are considering? *If 'yes' proceed, if 'no' further explain, and then repeat understanding check, until 'yes', then proceed.*

<u>Treatments for foot rot.</u>

We are only going to consider two treatments for footrot.

1. Intra-muscular injection of **long-acting** oxytetracycline antibiotic, correctly dosed for the weight of animal, plus topical oxytetracycline spray.
2. Foot trimming by someone who is proficient and experienced at trimming, plus topical oxytetracycline spray.

No other treatments will be given.

Do you have any questions concerning the treatments we are considering? *If 'no' proceed, if 'yes' clarify, repeat question until 'no', then proceed.*

Our question concerns **<u>ewes</u>** that are lame in **one foot** due to footrot, and we are interested in how likely you think it is that they will be cured from their lameness within the **first 5 days** after receiving one of these 2 treatments. By 'cured' we mean that the ewes will not have an observable limp and will not be head-flicking. You do not know how long the ewes have been lame for, but it is unlikely that they will have been lame for more than 2 weeks.

There could be several factors that you may believe will influence the cure rate in the first 5 days, other than which treatment was given; for example, you may believe that cure rates would be poorer in ewes that had more severe lameness to start with and so forth.

However for this question we are <u>not</u> interested in your opinion about how these factors will affect the outcome. All we are interested in is your opinion about any difference in the cure rates that is directly attributable to which treatment was given; in other words, you can assume that the appropriate corrections have been made for all factors that can possibly influence the cure rates <u>except</u> which treatment was given, so this is the only thing you need to think about.

Please also note (although it may sound obvious!) that we are asking for your opinion about what the <u>actual</u> answer is, such that this is the answer that would emerge after we have robustly compared the two treatments scientifically, and as a result we are absolutely certain we know what the actual answer is. *[Statement to make clear the question concerns an epistemic quantity and to avoid participants including aleatory uncertainty in their answers]*

Naturally you are not exactly sure what the actual answer is. But presumably you think some answers are going to be more likely than others. As we explained with the examples from earlier, we have given you 20 chips, each worth a '5% chance'. On the sheet, we ask you to consider each of the intervals of difference shown, and place chips in the intervals to indicate your **weight of belief** that the difference in the cure rates will fall in a given interval.

Researcher checks that vet understands how to fill the sheet in, and explains what their answers means, by feeding back some inferred probabilities, particularly the plausible range, and mode.

By placing the chips on the sheet, you have created a shape that represents how plausible you think it is that the actual difference in the cure rates (due to which treatment was given) will turn out to fall in the given intervals. Please now take time to have a final look at the <u>shape</u> and <u>distribution</u> of your chips and check that this is what you really think and how confident you are; if you want to change your mind and move any of the chips about, please do so. Note: The chips are easily removable so if you change your mind you can re-position them very easily.

If participant re-adjusts any chips, facilitator to feedback some inferred probabilities, particularly the plausible range, and mode, and then re-read the above paragraph.

<u>Baseline cure rates with each treatment and range</u>

Please now think **only** about the treatment 'intra-muscular long-acting oxytetracycline and topical oxytetracycline spray'. For a typical group of ewes, lame with footrot in one foot, what % of them would you <u>expect</u> to cure within 5 days of receiving this treatment? (just give one figure): X=………

You may have some uncertainty about this figure. You may believe that the cure rate could in fact turn out to be lower or higher than this. Please indicate a **lower** and an **upper** boundary for this figure, such that you believe there is very little chance that the cure rate could fall outside of this range…………..

Please now think only about the treatment 'foot trimming plus topical oxytetracycline spray'. For a typical group of ewes, lame with footrot in one foot, what % of them would you expect to cure within 5 days of receiving this treatment? (just give one figure): Y=……

You may have some uncertainty about this figure. You may believe that the cure rate could in fact turn out to be lower or higher than this. Please indicate a **lower** and an **upper** boundary for this figure, such that you believe there is very little chance that the cure rate could fall outside of this range……………..

Coherency check - 1: Researcher calculates Z=X-Y and reports this to the vet. Researcher to ensure Z has been given at least some weight of belief on the elicitation sheet. *If not, researcher to check vet understands the questions, and invite the vet to revise their answers, if needed.*

Coherency check – 2: Once all other factors that could affect the cure rates have been taken into account, do you think that one treatment is definitely better than the other? *Researcher to check this answer agrees with their previous answers.*
