# Supplementary material for: How Does Reviewing the Evidence Change Veterinary Surgeons’ Beliefs Regarding the Treatment of Ovine Footrot? A Quantitative and Qualitative Study
Source: PLoS One. 2013 May 16;8(5):e64175. doi: 10.1371/journal.pone.0064175 (PMC3655936; doi:10.1371/journal.pone.0064175)
Supplement: Appendix S2 — Schematic illustration of the 6 training examples provided. (PDF) [file pone.0064175.s002.pdf]

Example 1

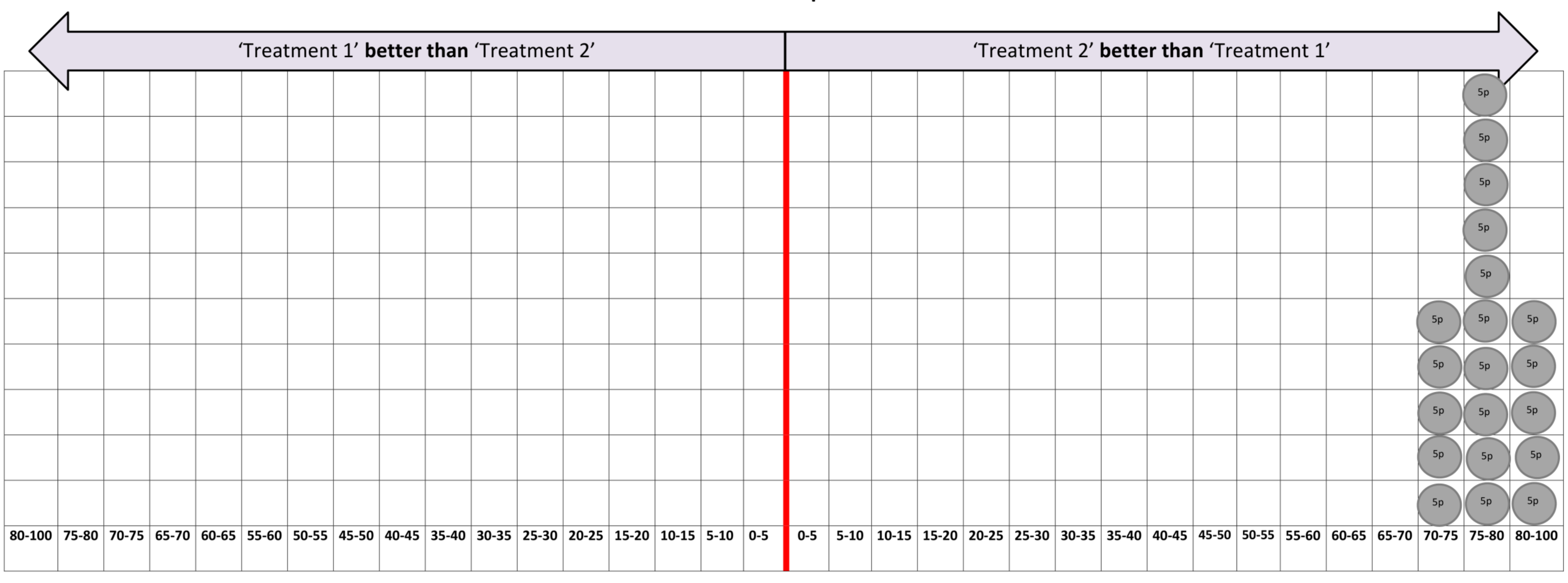

The **difference** between the cure rates (%)

Example 2

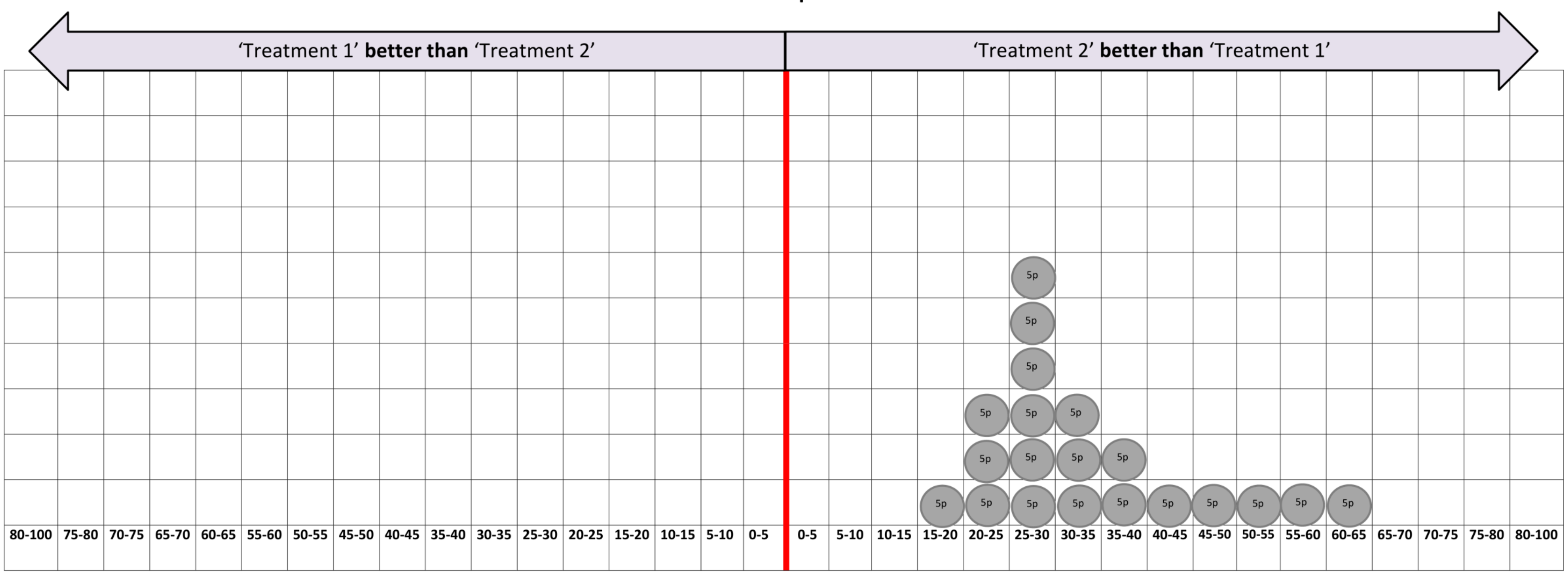

The **difference** between the cure rates (%)

Example 3

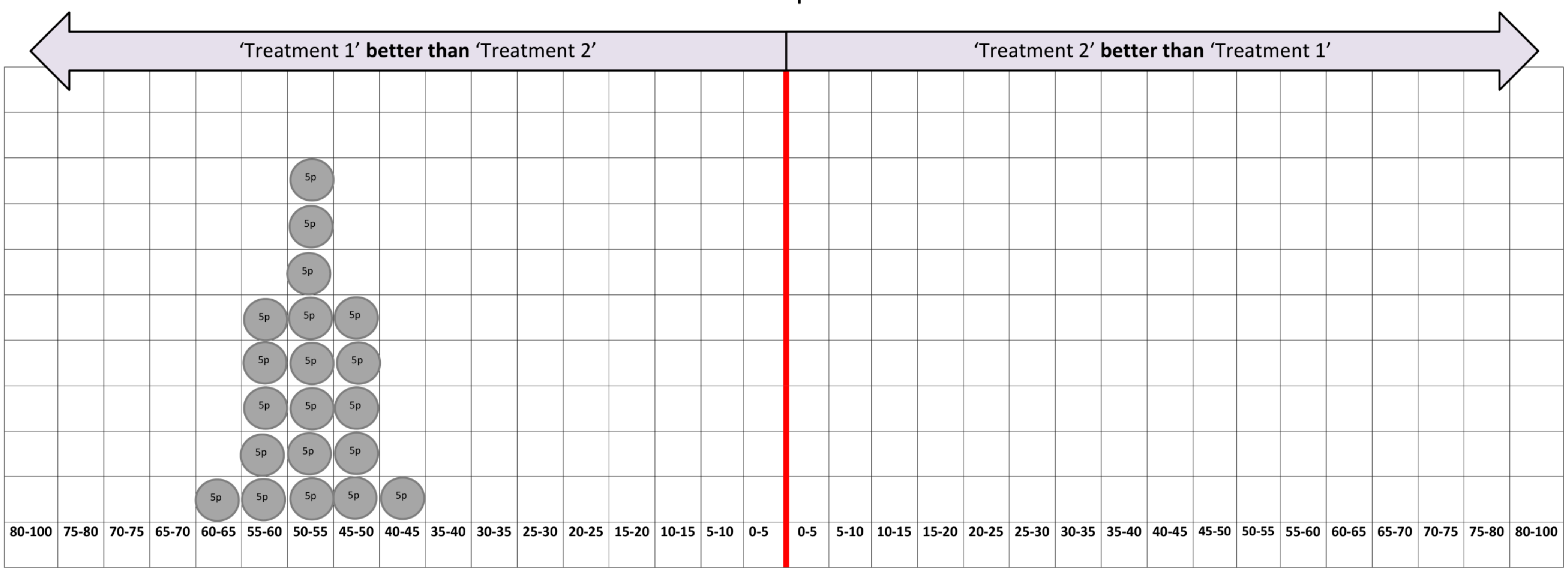

The **difference** between the cure rates (%)

Example 4

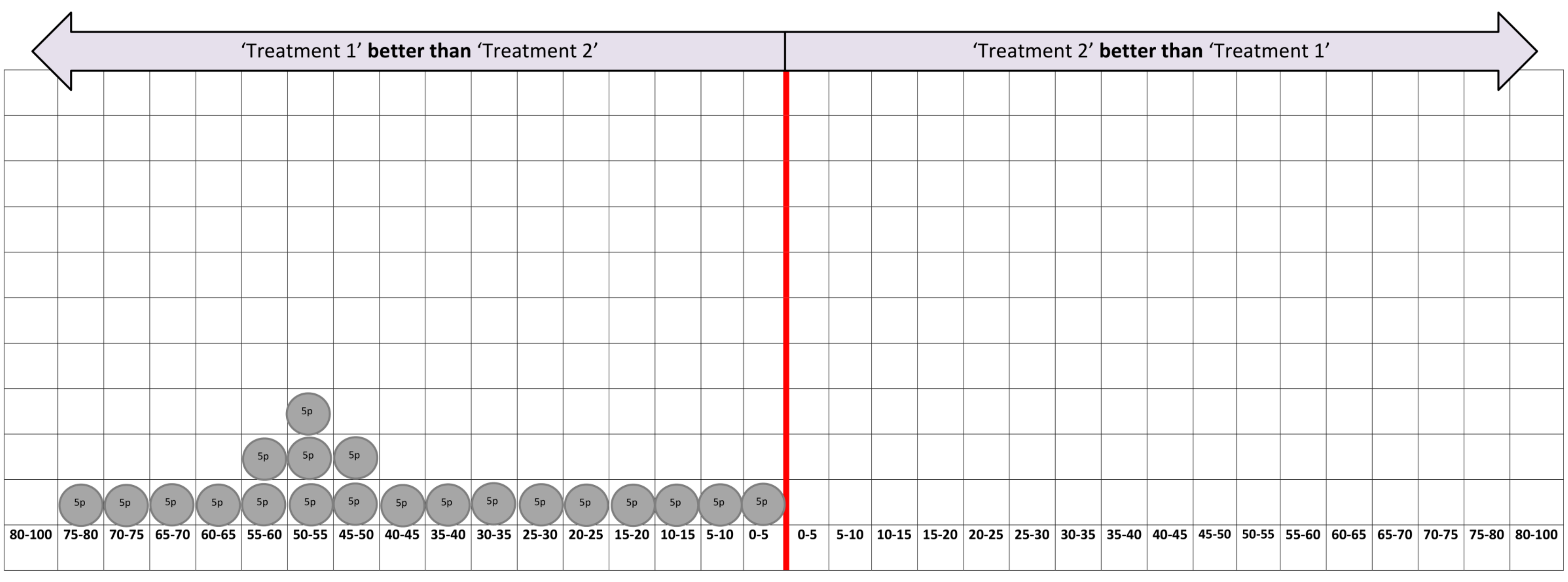

The **difference** between the cure rates (%)

Example 5

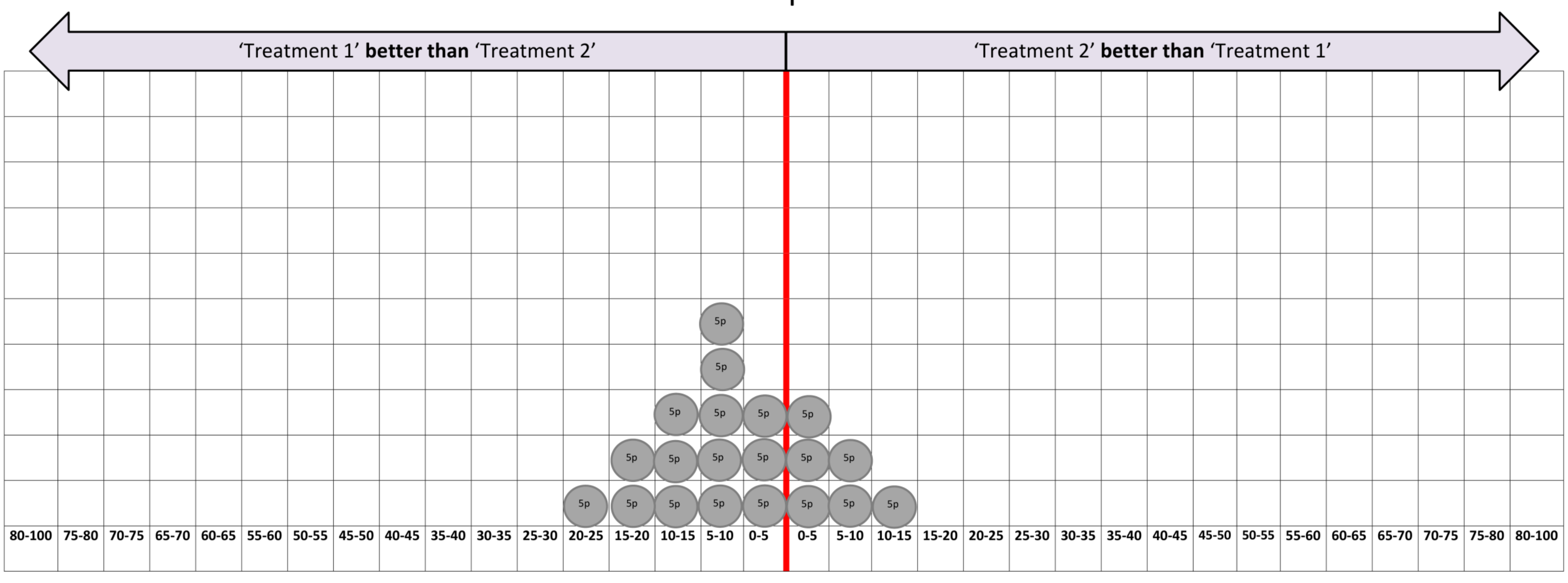

The **difference** between the cure rates (%)

Example 6

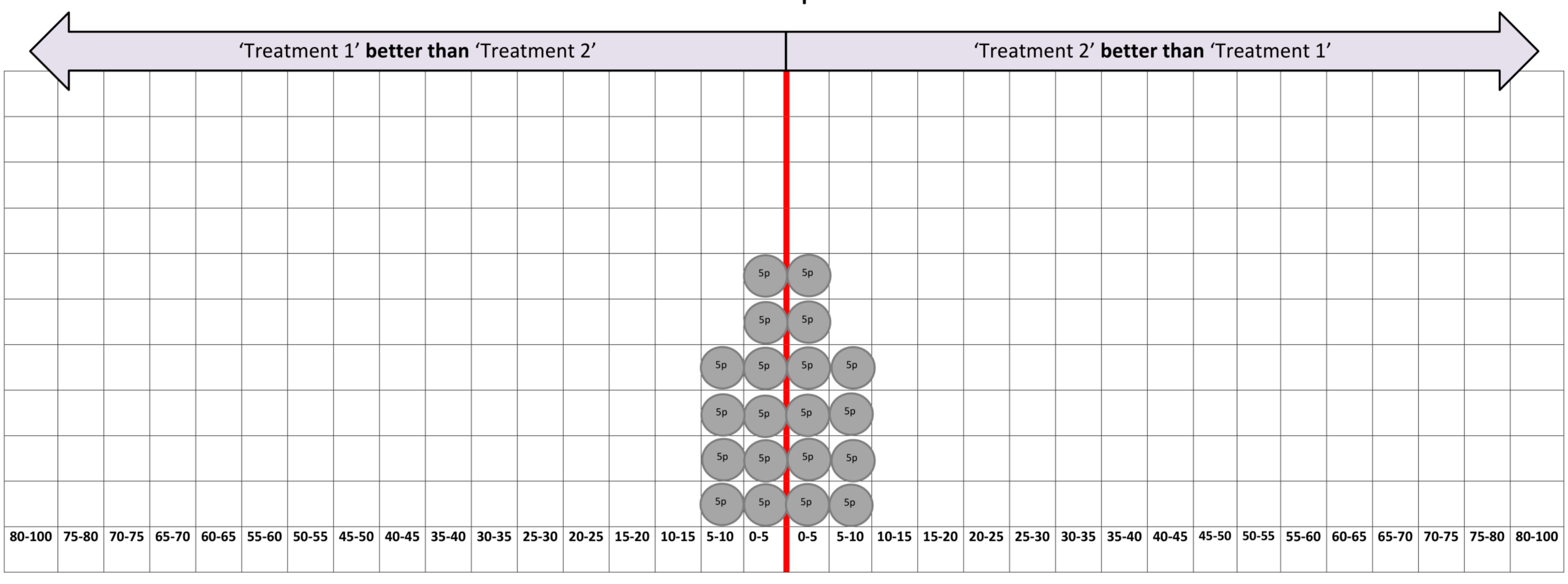

The **difference** between the cure rates (%)
