# Supplementary figures and images for: How Does Reviewing the Evidence Change Veterinary Surgeons’ Beliefs Regarding the Treatment of Ovine Footrot? A Quantitative and Qualitative Study
Source: PLoS One. 2013 May 16;8(5):e64175. doi: 10.1371/journal.pone.0064175 (PMC3655936; doi:10.1371/journal.pone.0064175)

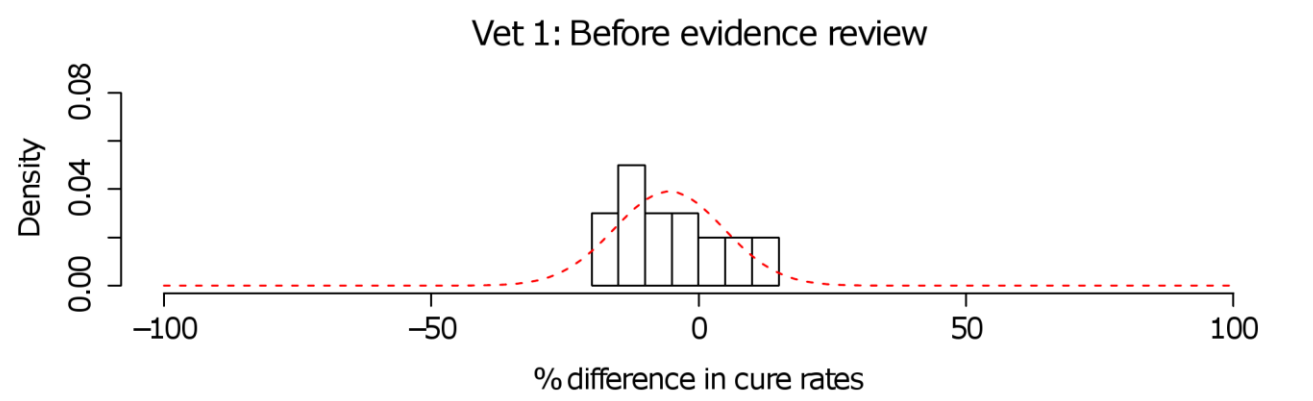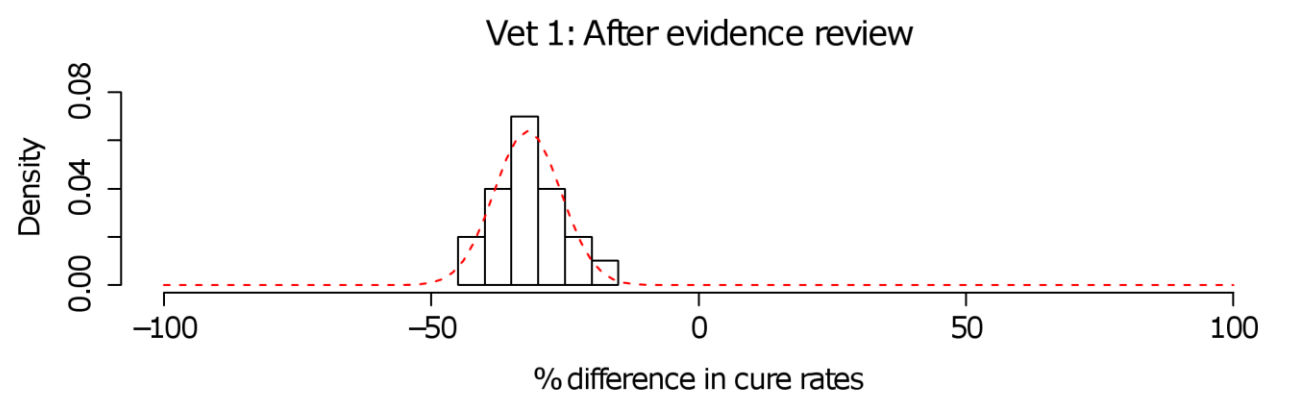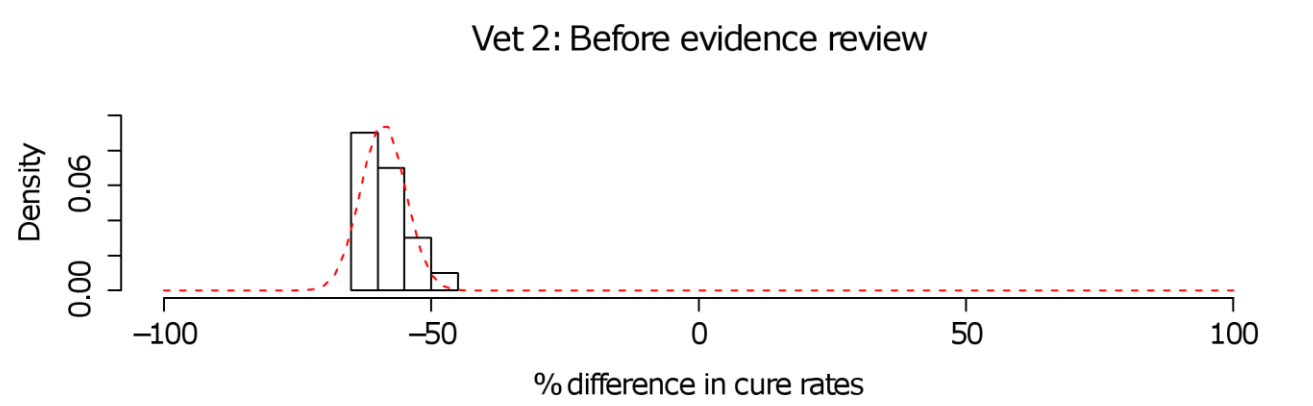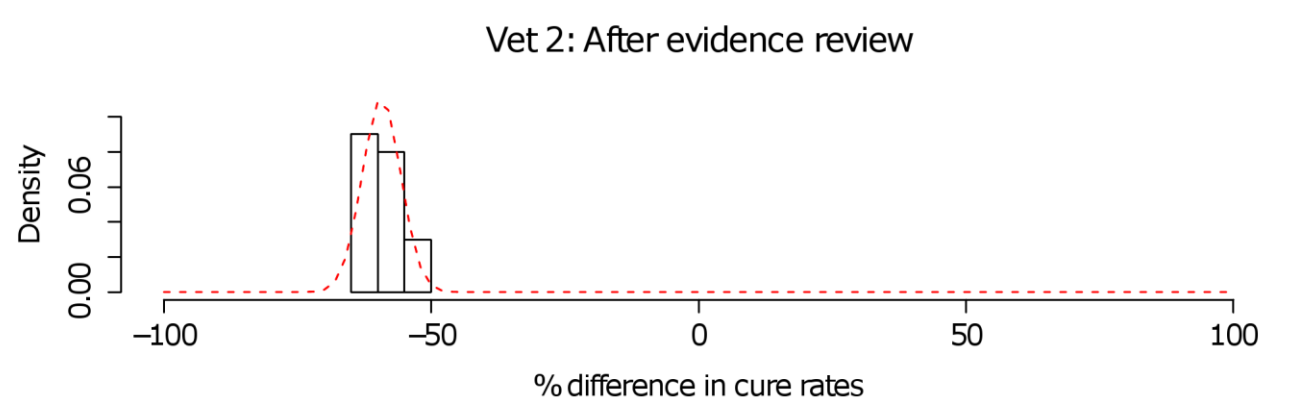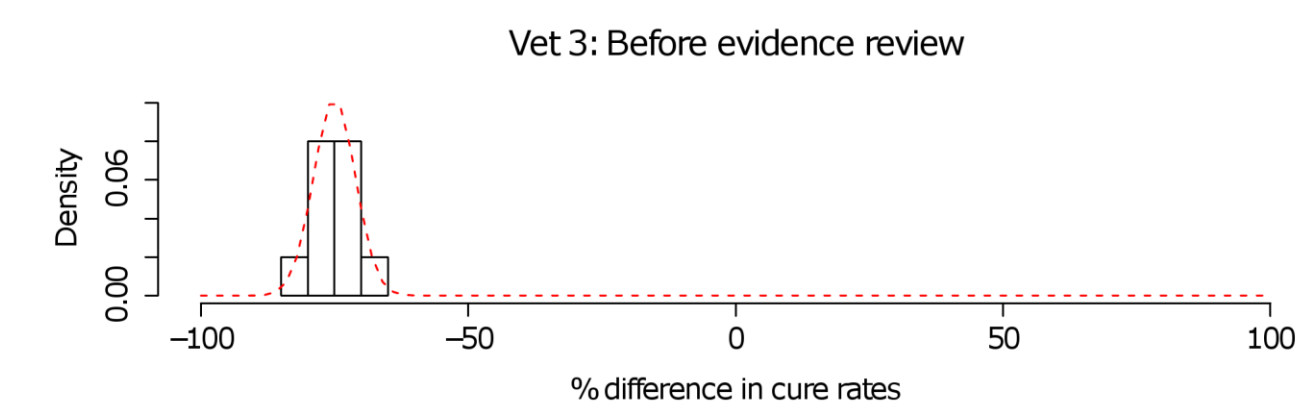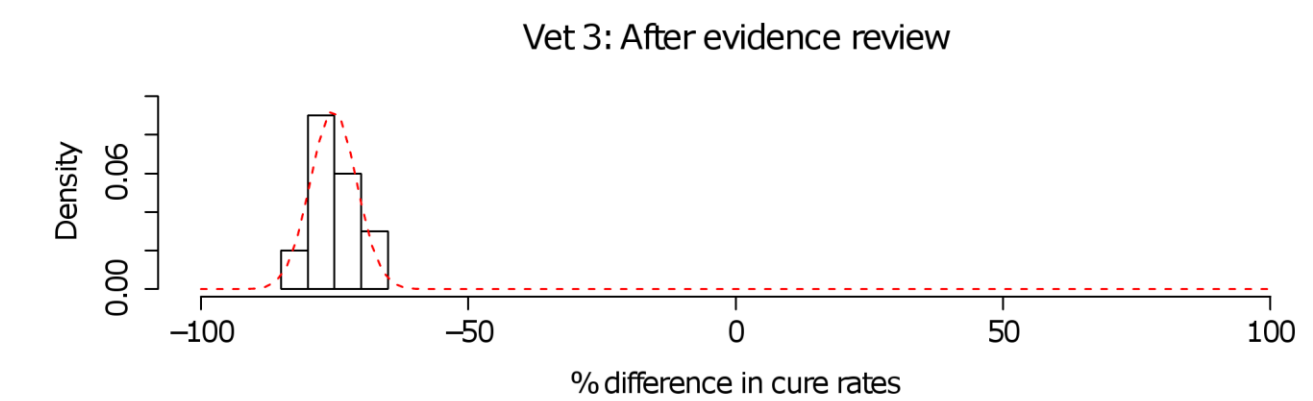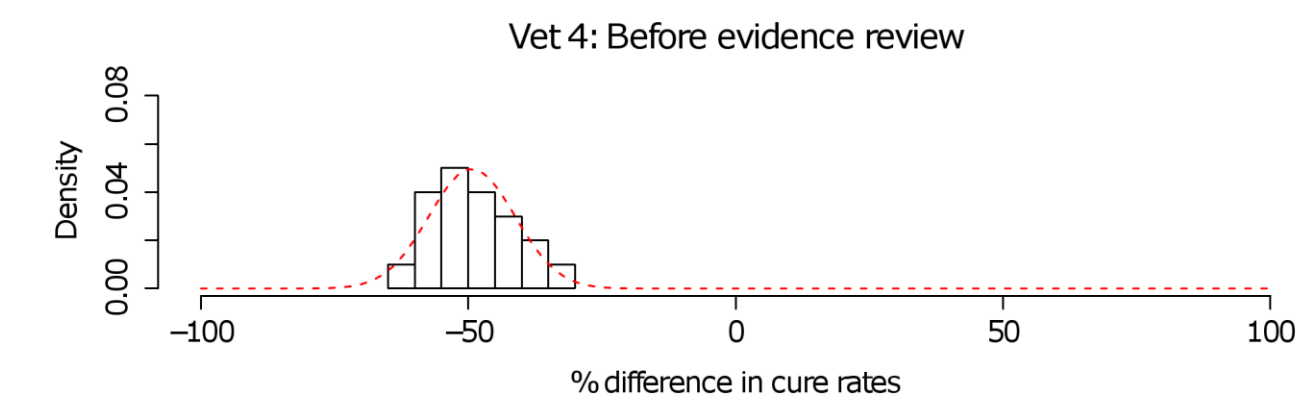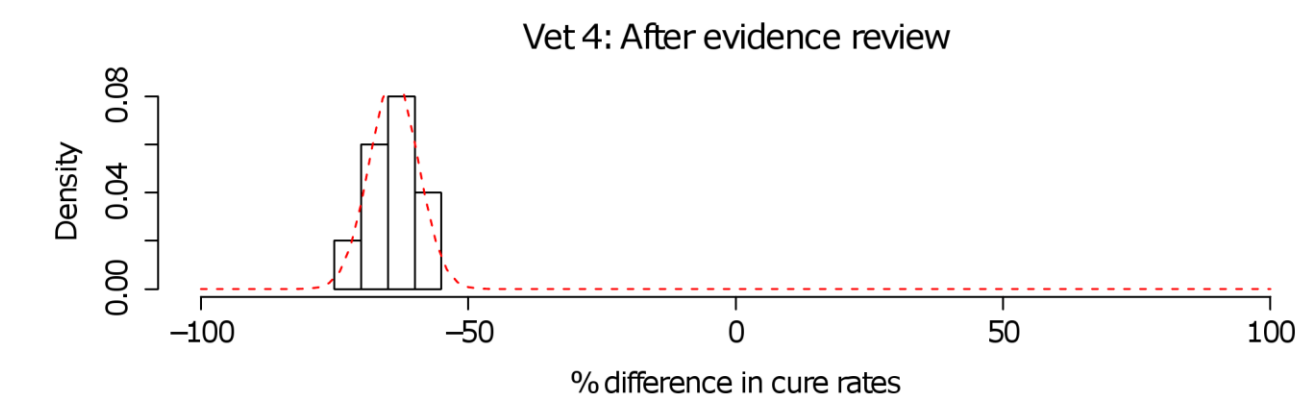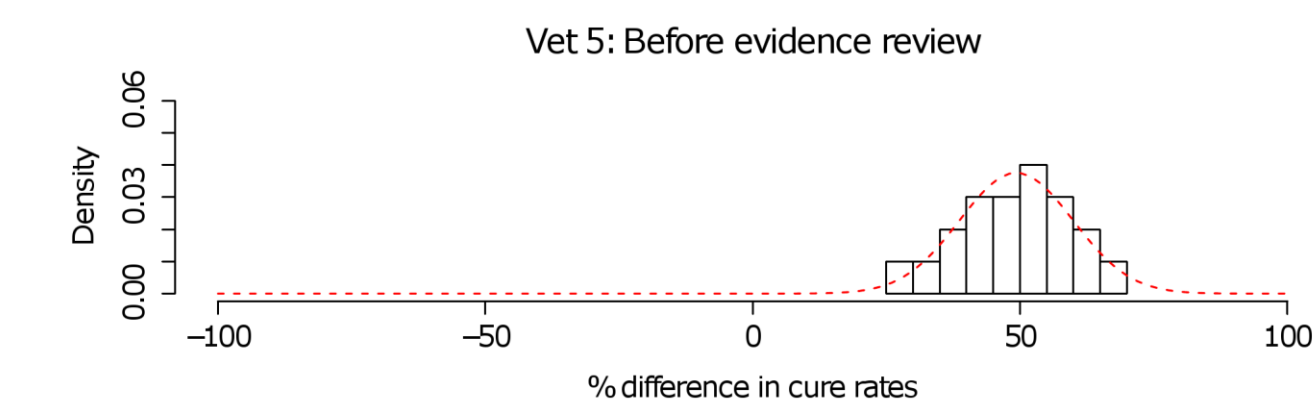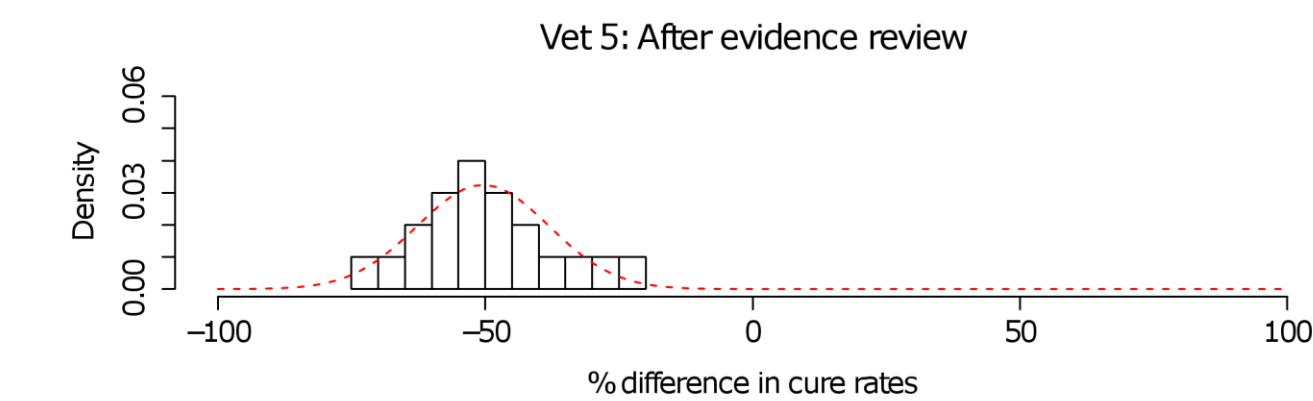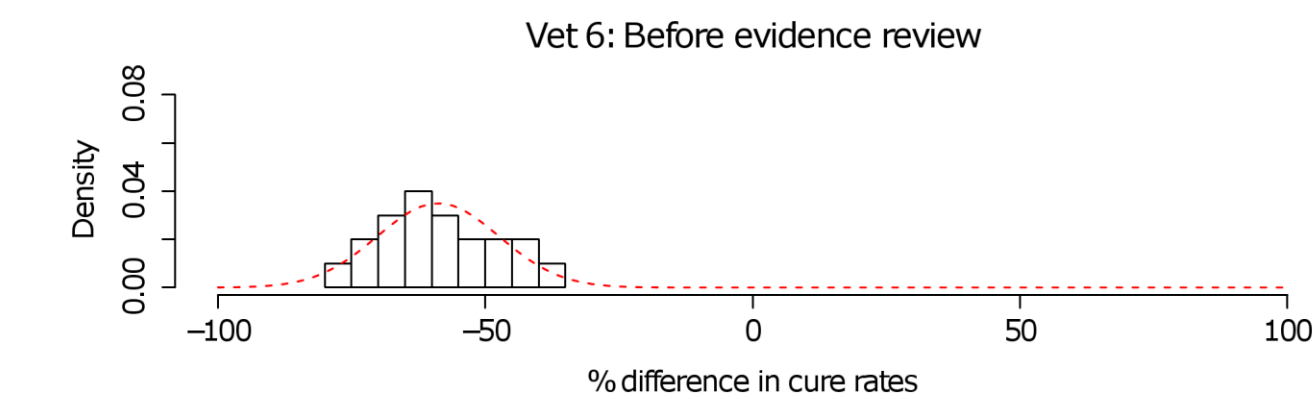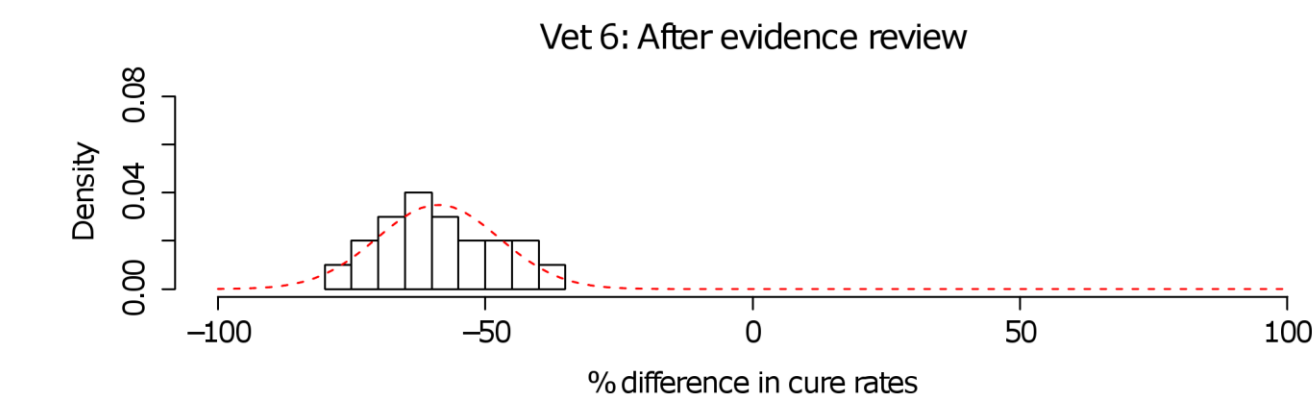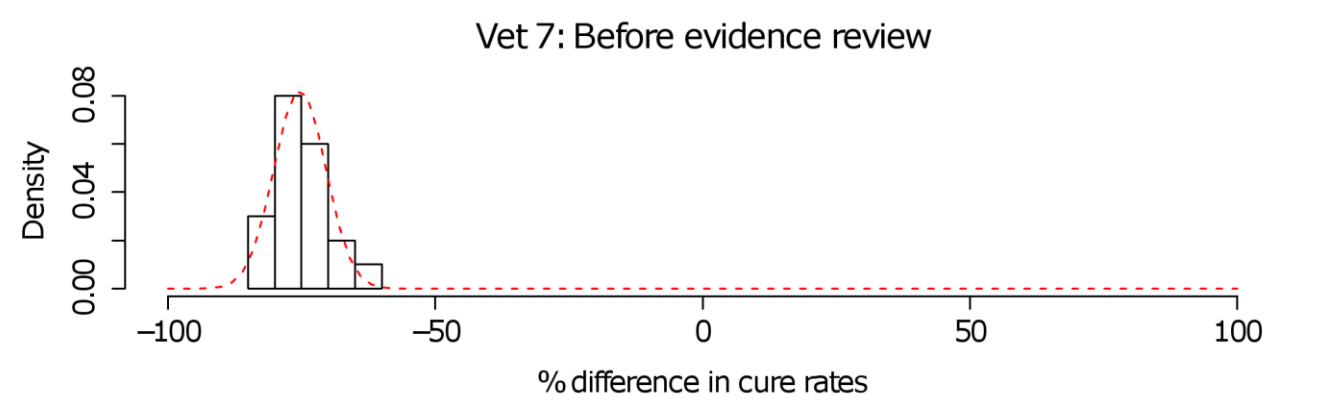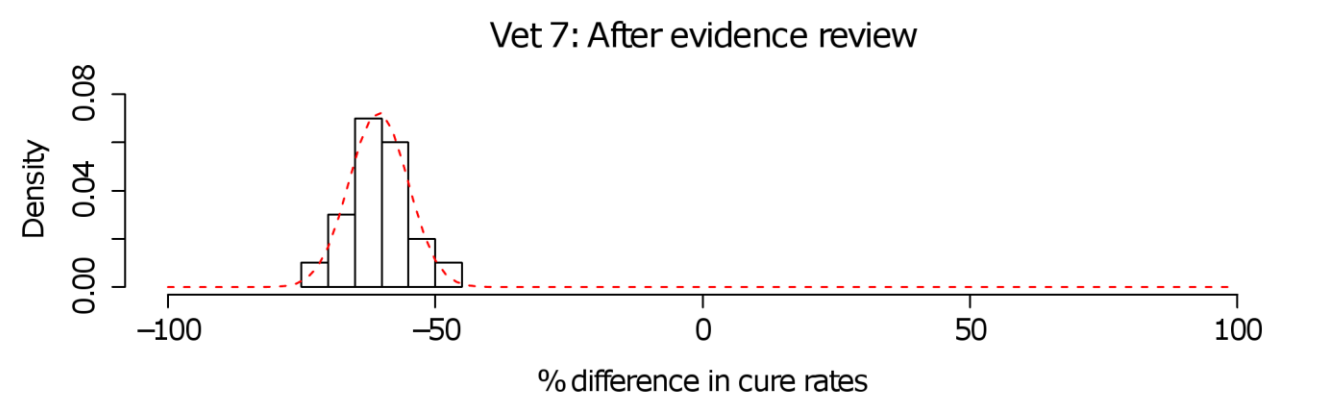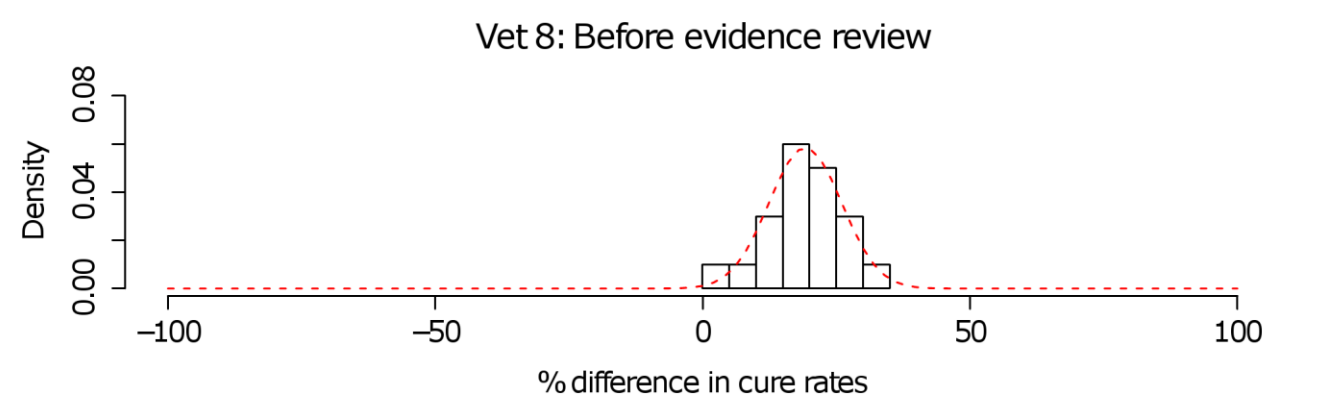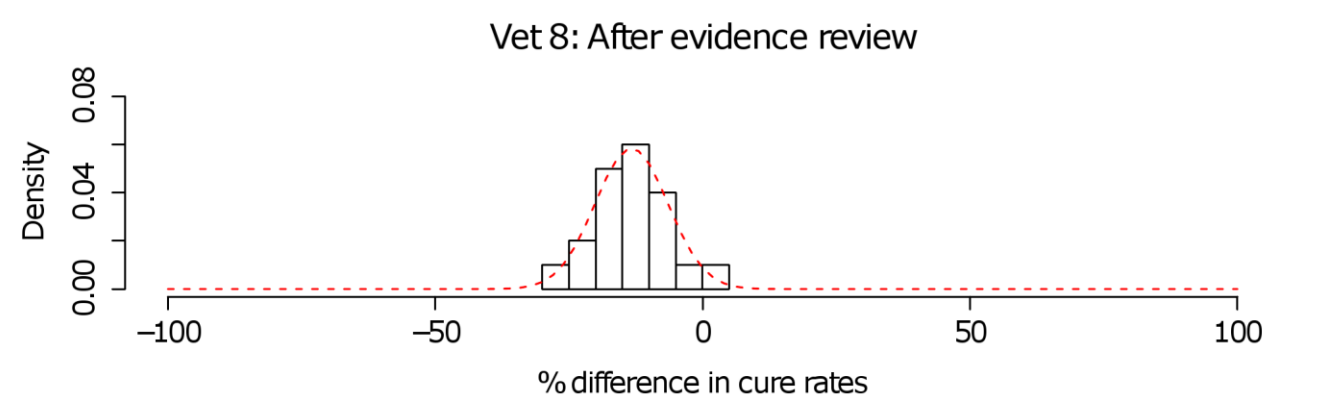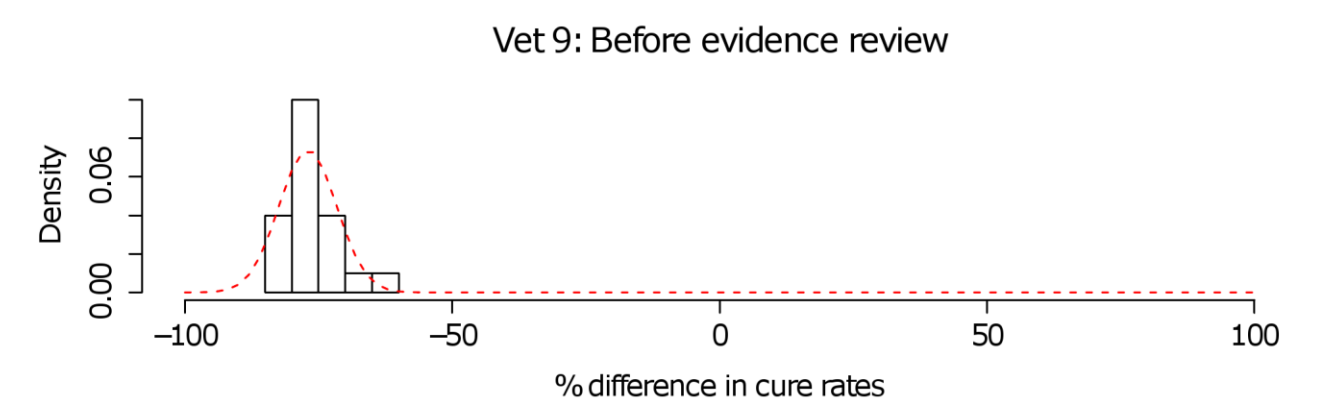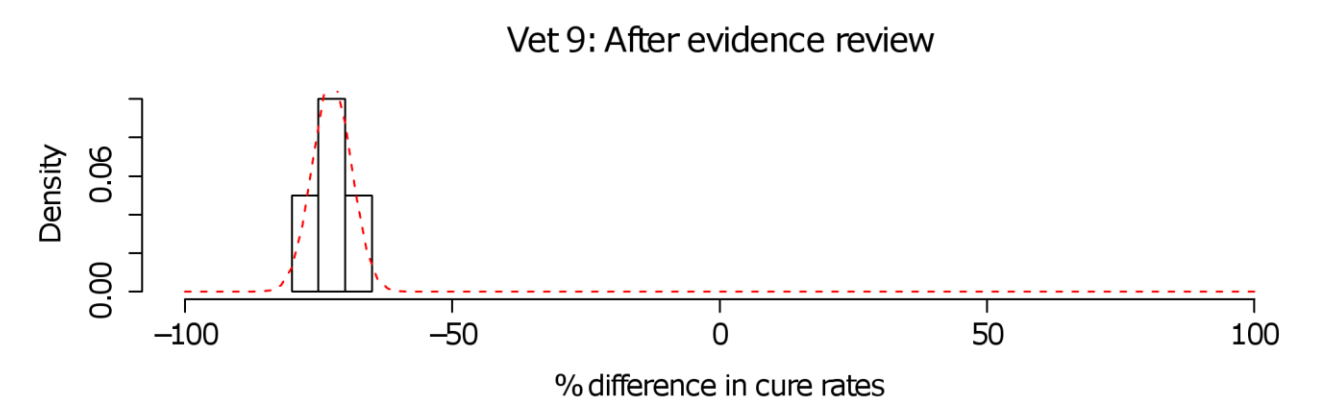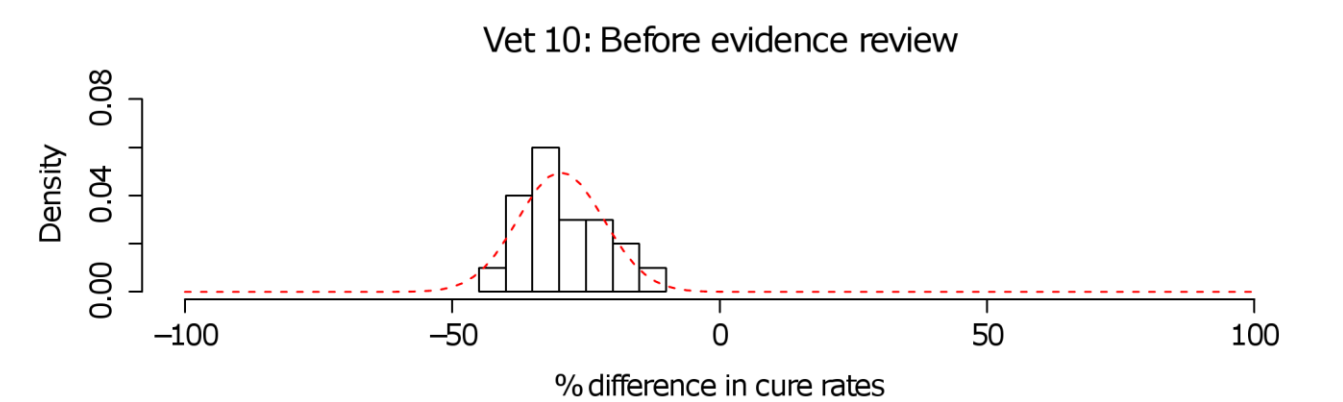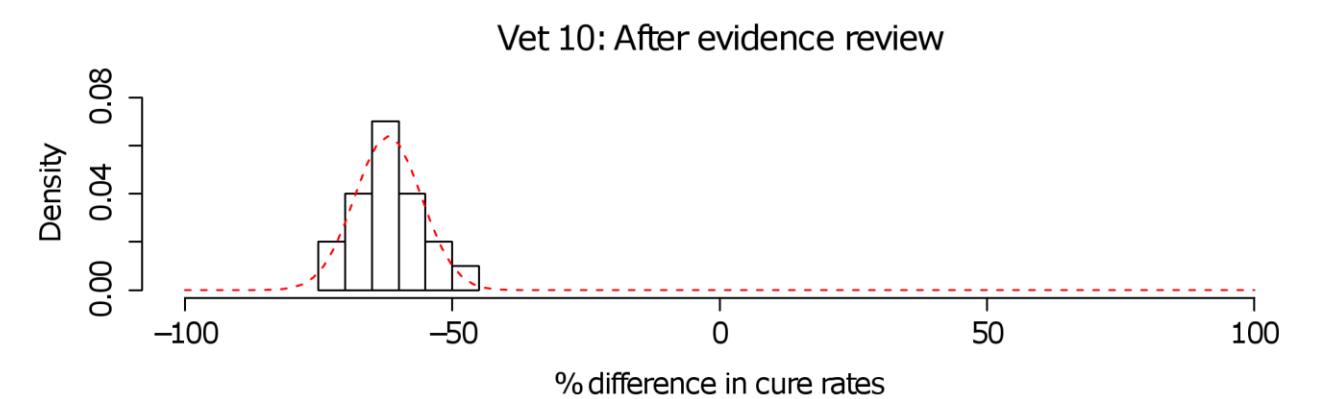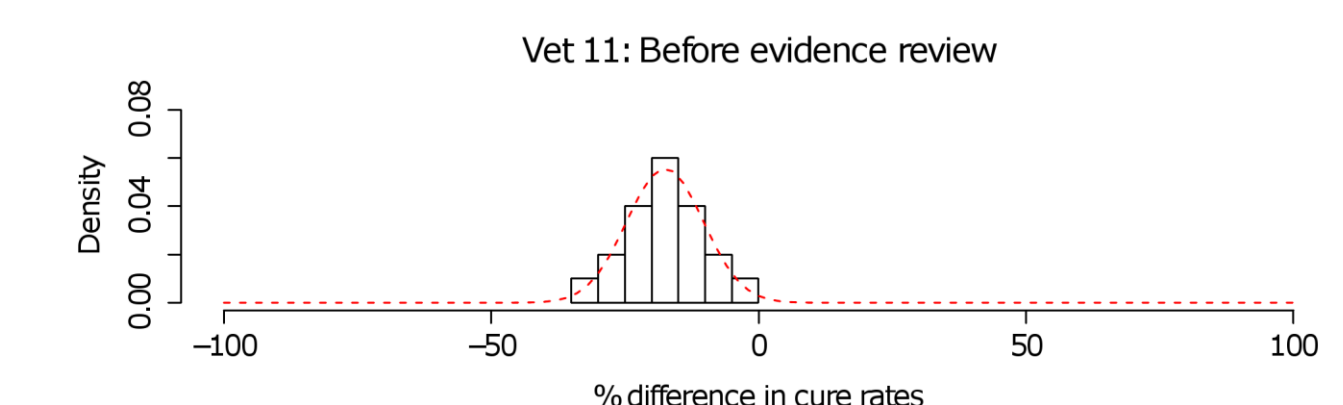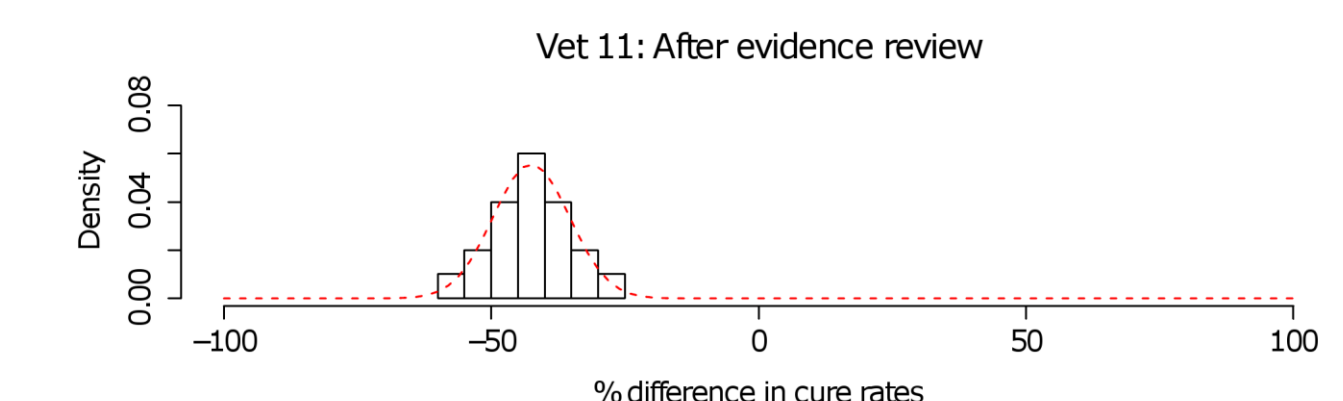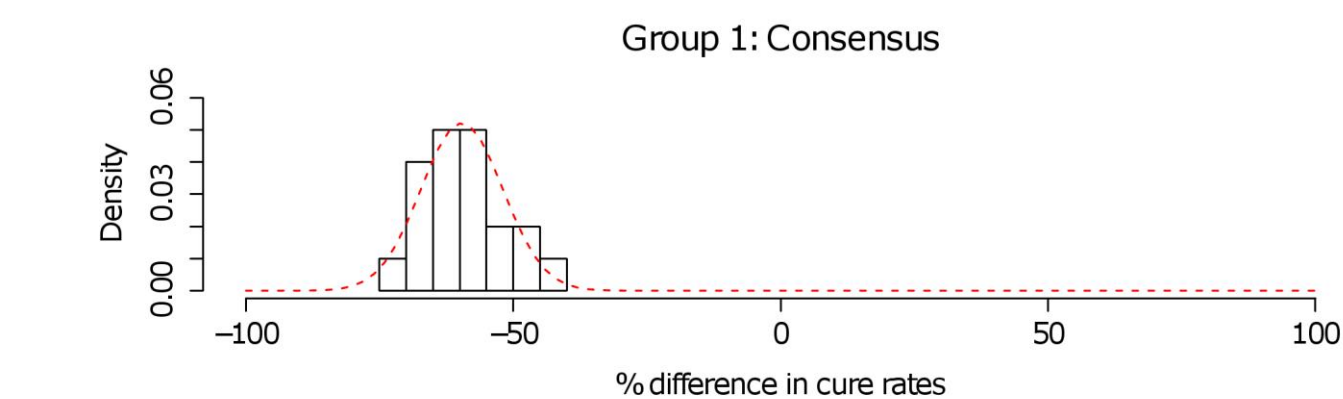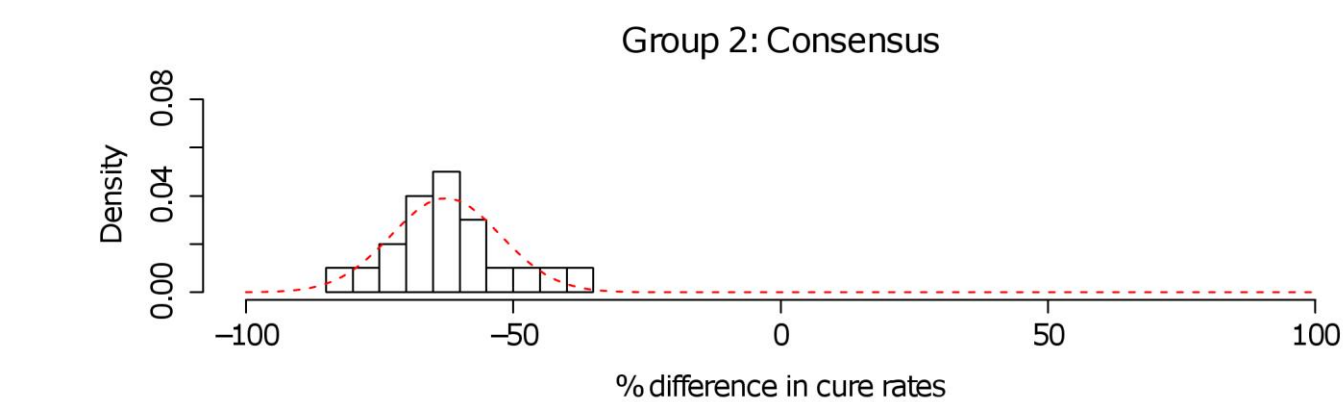

Supplement: Appendix S3 — Gaussian probability distributions fitted to the raw elicitation data. (PDF) [file pone.0064175.s003.pdf]
