# Supplementary material for: How Does Reviewing the Evidence Change Veterinary Surgeons’ Beliefs Regarding the Treatment of Ovine Footrot? A Quantitative and Qualitative Study
Source: PLoS One. 2013 May 16;8(5):e64175. doi: 10.1371/journal.pone.0064175 (PMC3655936; doi:10.1371/journal.pone.0064175)
Supplement: Appendix S4 — Examples given by veterinary surgeons of tailoring their veterinary advice. (PDF) [file pone.0064175.s004.pdf]

Appendix S4: Examples of scenarios where practitioners cited tailoring their veterinary advice to the clinical situation

1. Tailoring advice for welfare concerns:

*"If I know they're a farmer that isn't brilliant in terms of welfare or quite often has the odd lame sheep, lame cow wandering around and isn't doing anything about it then I'll be a bit more pushy about following up and calling them back."*

2. Tailoring advice according to strongly held farmer beliefs regarding treatment:

*"Yes, because I know with some of them they will not accept they do not trim. So you say, 'Well OK, trim a few flappy bits off.'... ... But if you've got a piece hanging off like that you cannot convince the farmer that that just needs to be left and it'll be perfectly OK. So you have to say... 'OK, just cut the big flappy bit off, don't go anywhere near the actual foot, just take the hangnail off and try and keep away from anything that might be sensitive tissue.'"*

3. Tailoring advice because of concerns it will not be implemented correctly and therefore fail to work:

*"...the whole vaccination thing, the reason I didn't jump in to say that is because a lot don't do it properly and then it doesn't end up working, which probably puts me off recommending it as a start – on the good farms, yes, I would get them going on the vaccine, but again, stressing that they have to do it properly and all the rest of it."*

4. Tailoring advice by delaying treatment in favour of completing the task properly:

*"I mean, as sad as it is, you do have to be a bit practical about it and there isn't always the farm labour and even if it means doing as much with the ewes but sort of splitting them into groups, so you'd say, 'Do however many ewes that day if you can cope with that, and then that', rather than, 'Do them all but don't do a proper job!' <laughs> ... do you know what I mean? ... you're almost best to have the others waiting a day and then do a proper job on them, than saying, 'Do all that with them now', you just have to work around it a bit."*

5. Tailoring advice associated with the knowledge and abilities of the farmer:

- *"It does vary dramatically [depending] on the knowledge and the experience of the farmer. If you've got somebody that's to be honest, hopeless with them, then it might sometimes be a case of getting them to bring the animal to us if it's a single or small number of animals affected. Or in some cases we might insist on going out and seeing them. With the large commercial guys who are very experienced sometimes the advice we give would be over the phone."*

- *"... a lot of smaller flocks are run by an ageing population of sheep farmers and catching and turning them is not necessarily an option for them and in which case we*

*might suggest putting them through a footbath and injecting the lame ones, rather than turning them over and trimming them out."*

6. Tailoring advice associated with size of flock and facilities available:

- *"Yeah, well, sometimes I'll tell them ... it all depends on how severe they are and such-like but sometimes I'll tell them, if they've got a big flock, you know, of a couple of thousand sheep, and there's maybe 300-400 lame, then I'll tell them, 'Right, footrot; vaccinate everything and then two weeks later if anything's still lame, inject with antibiotics at that stage', because the footrot vaccine does actually help clear them up anyway and realistically they're not going to be turning that many sheep over to see if they're actually lame. The other thing I think is quite a good idea is to footbath them as a way of finding out if they're lame, so if they run them up the runner and put them through the ... because often when sheep are in the pens, it's hard to tell if there lame or not because they're running at speed to try and get away, but out in the field you see the lame ones hobbling around and the farmer, he gathers them in the pens and it can be quite hard to see if they're lame but if they're footbathed, it makes it hurt a bit more and then you can pick them out and get a better idea of how many are lame."*

- *"On a farm with an active shepherd and a good dog, I would advise him to catch sheep in the field and treat them and mark them on a day-by-day basis. On a farm that's running a thousand ewes in a big park field, that's not feasible, so we would suggest gathering them weekly in the field, in mobile pens, so they're not coming in to a communal yard; they're just gathered in a corner and, you know, try and spot lame ewes, so that they can be treated once a week in groups and that's more likely to be effective than trying to chase them in a hundred acre paddock! So yes."*

- *"Yeah, I mean if I know they're not going to inject them all them sometimes I'll go down the footbath route more sooner than the injection route. It just depends what facilities they've got and normally we have a bit of a discussion about what facilities they've got, what handling facilities they've got."*

- *"And often part of the advice will be upgrading their facilities or getting mobile pens ... because quite a few of the facilities basically just spread footrot every time they gather the sheep. So yeah, I mean, there's quite a few, you try and get them to get more gravel in or put some concrete down or elevate the belts or whatever they have to try and ... so they're not standing sheep in a claggy mess and putting them out through a different gate and things."*

- *"...and the biggest kind of bug bear I have is when I have conversations with people about foot bathing and they're talking to me that they've foot bathed and that hasn't potentially worked, and we're therefore ... I always ... did you rest them on hard standing afterwards or did you run them through the footbath and ran them straight into the soggy wet field? Oh, I ran them straight into the soggy wet field. I know I should put them on hard standing, but I haven't got any. You're always facing issues*

*where … I would say, 'Is there any way … your treatment is not going to be effective if you can't do it effectively. Is there any way we can do anything? You might be potentially wasting your money by doing that. We've got to either think of something else to do or you've got to look at another way around it because there's no point in doing that if it's just going to get washed straight off' if that makes sense. So I think you've always got to be practical, like there's an ideal scenario of advice and treatment, and there's the practical world where … and you will have very different clients that you know. If you know your clients, like I know a lot of my sheep clients pretty well now and I know who will follow my advice to the letter, who will need advice tailored to make sure they do the best that they can, but it won't necessarily be the ideal protocol if you see what I mean."*
